# Autonomous action and cooperativity between the ONECUT2 transcription factor and its 3′ untranslated region

**DOI:** 10.3389/fcell.2023.1206259

**Published:** 2023-07-05

**Authors:** Kenneth Steadman, Sungyong You, Dustin V. Srinivas, Lila Mouakkad, Yiwu Yan, Minhyung Kim, Smrruthi V. Venugopal, Hisashi Tanaka, Michael R. Freeman

**Affiliations:** Division of Cancer Biology and Therapeutics, Biomedical Sciences and Pathology and Laboratory Medicine, Department of Urology, Cedars-Sinai Medical Center, Samuel Oschin Comprehensive Cancer Institute, Los Angeles, CA, United States

**Keywords:** competitive endogenous RNA network, neuroendocrine prostate cancer, polycomb repressing complex, androgen metabolism, prostate cancer

## Abstract

The transcription factor ONECUT2 (OC2) is a master transcriptional regulator operating in metastatic castration-resistant prostate cancer that suppresses androgen receptor activity and promotes neural differentiation and tumor cell survival. OC2 mRNA possesses an unusually long (14,575 nt), evolutionarily conserved 3′ untranslated region (3′ UTR) with many microRNA binding sites, including up to 26 miR-9 sites. This is notable because miR-9 targets many of the same genes regulated by the OC2 protein. Paradoxically, OC2 expression is high in tissues with high miR-9 expression. The length and complex secondary structure of OC2 mRNA suggests that it is a potent master competing endogenous RNA (ceRNA) capable of sequestering miRNAs. Here, we describe a novel role for OC2 3′ UTR in lethal prostate cancer consistent with a function as a ceRNA. A plausible ceRNA network in OC2-driven tumors was constructed computationally and then confirmed in prostate cancer cell lines. Genes regulated by OC2 3′ UTR exhibited high overlap (up to 45%) with genes driven by the overexpression of the OC2 protein in the absence of 3′ UTR, indicating a cooperative functional relationship between the OC2 protein and its 3′ UTR. These overlapping networks suggest an evolutionarily conserved mechanism to reinforce OC2 transcription by protection of OC2-regulated mRNAs from miRNA suppression. Both the protein and 3′ UTR showed increased polycomb-repressive complex activity. The expression of OC2 3′ UTR mRNA alone (without protein) dramatically increased the metastatic potential by *in vitro* assays. Additionally, OC2 3′ UTR increased the expression of Aldo-Keto reductase and UDP-glucuronyl transferase family genes responsible for altering the androgen synthesis pathway. ONECUT2 represents the first-described dual-modality transcript that operates as both a key transcription factor driving castration-resistant prostate cancer and a master ceRNA that promotes and protects the same transcriptional network.

## 1 Introduction

3′ UTR lengths of gene orthologs have consistently increased throughout evolution (Mazumder et al., 2003)*.* The global tissue patterns for specific 3 ′UTR lengths has been shown across many species and controls developmental patterns in mice (Ji et al., 2009; Ulitsky et al., 2011) and *Drosophila* (Smibert et al., 2012)*.* Ubiquitously expressed genes have elongated 3′ UTRs when expressed in neural tissues (Wang & Yi, 2014)*.* Miura et al. (2013) analyzed 2,035 3′ UTR extensions in mice and found that the miRNA-binding sites that increased the most in longer 3′ UTRs were those known to regulate neural pathways. These neural-acting miRNAs included miR-9, miR-96, miR-124, miR-125, and miR-137*.* Cancer cells use alternative polyadenylation and cleavage to shorten 3′ UTRs, which activate oncogenes without genetic mutation (Mayr and Bartel, 2009). A disrupted competitive endogenous RNA (ceRNA) oncogene network in prostate cancer was associated with higher risk; this subnetwork was shown to be driven by 3’ UTR shortening (Li et al., 2014)*.*

OC2 was identified as a master regulator of androgen receptor (AR) networks in metastatic castration-resistant prostate cancer (mCRPC) (Rotinen et al., 2018)*.* OC2 interferes with AR programming by direct regulation of AR network genes (Rotinen et al., 2018)*.* OC2 induces a transcriptional network that drives neural differentiation and lethal neuroendocrine prostate cancer (NEPC (Rotinen et al., 2018 and Guo et al., 2019). Neuroendocrine prostate cancer (NEPC) is characterized by a strong reduction of AR programming during transdifferentiation from adenocarcinoma into neuroendocrine-like cells, in response to treatment by antiandrogen therapies (Guo et al., 2019). Only 1% of primary prostate tumors are considered to be of the neuroendocrine phenotype, but they make up 25% of lethal mCRPC (Gupta & Gupta, 2017). NEPC is associated with frequent visceral metastases and decreased PSA levels (Conteduca et al., 2014). Most importantly, OC2 activity in these scenarios corresponds to poor patient outcomes. However in breast cancer, a report that showed repression of OC2 by miR-9, miR-195, and miR-203 resulted in an increase of stemness (Shen et al., 2019)*.*

The PcG (polycomb group) proteins form complexes called polycomb-repressive complexes (PRCs). PRC2 contains EZH2, EED, SUZ12, and RbAp48 (Levine et al., 2004). The PRCs act as histone methyltransferases to tri-methylate the histone H3 protein. This H3K27 methylation causes epigenetic silencing of chromatin and represses gene transcription.

The PRC family proteins were found to be specifically upregulated in NEPC (Clermont et al., 2015). Multiple datasets from clinical and xenograft tumor NEPC tissues showed that EZH2 and CBX were the most enhanced epigenetic regulators. EZH2 was previously shown to regulate neuronal differentiation and neurodevelopment (Di Meglio et al., 2013; (Hirabayashi et al., 2009). EZH2 also acts in a PRC-independent manner. PRC-independent activity methylates REST, modifying its stability (Lee et al., 2018)*.* This decrease in REST stability maximizes chromatin accessibility for neuronal differentiation (Lee et al., 2018). The role of EZH2 as an oncogenic driver is clear in prostate cancer. EZH2 is involved in the progression to a lethal disease and is a prognostic biomarker (Varambally et al., 2002)*.* EZH2 is a protein, which has functional activity important to both neurogenesis and aggressive prostate cancer. The goal of this study was to understand the role of the very long 3’ UTR of the OC2 transcript in aggressive prostate cancer, in particular the functional capacity of the OC2 mRNA as a ceRNA and how it interacts with the OC2 transcription factor protein.

## 2 Methods

### 2.1 Cell culture

22Rv1, C4-2, and LNCaP were obtained from ATCC and maintained in RPMI-1640 + L-Glutamine +25 mM HEPES (Gibco #22400-089) with 1% penicillin–streptomycin (Gibco #15140-122) and 10% fetal bovine serum heat inactivated (Hyclone #SH30071.03) at 37 C with 5% CO_2_.

### 2.2 Virus production

293T cells (ATCC) were plated onto 0.0005% poly-L-lysine-coated 6 cm dishes (Sigma P4832) and then transfected the following day using the JetPRIME transfection reagent (Polyplus) pMD2.G VSV-G envelope expressing plasmid (Addgene plasmid #12259), psPAX2 second-generation lentiviral packaging plasmid (Addgene plasmid #12260) along with the relevant experimental vector, and 5-uM chloroquine (InvivoGen #tlrl-chq).

### 2.3 MicroRNA transfections

Cells were transfected with synthetic miRNA mimics, miRNA inhibitor, and miRNA mimic negative controls using RNAifectin™ transfection reagent (Applied Biological Materials).

### 2.4 Northern blot analysis

A published protocol was used (He and Green, 2013). In brief, 15 microG of total RNA was mixed with 2x RNA-loading dye (New England Biolab), incubated at 65℃ for 20 min, and run on a denaturing RNA gel (1.2% agarose, 1x MOPS buffer) at 90 V for 5 h, along with the ssRNA ladder (New England Biolabs). The RNA was transferred onto a nylon membrane using Whatman TurboBlotter transfer system (GE Healthcare) according to the manufacturer’s instruction. The membrane was then UV crosslinked. DNA probes were prepared using the PCR DIG Probe system (Millipore Sigma). cDNA from a human cell line was amplified by PCR using the primers for ONECUT2 (5′ GAG​TCT​GCC​CAA​CTA​CGG​TC-3′ and 5′- GCG​TTT​GCA​CGC​TGC​C-3′) and GAPDH (5′-CAG​CCT​CAA​GAT​CAT​CAG​CAA​TG-3′ and 5′-AAA​TGA​GCT​TGA​CAA​AGT​GGT​CG-3′).

### 2.5 Microarray analysis

Gene expression was assessed by Agilent 60K microarray. RNA was extracted and then treated with DNAse to remove genomic DNA contamination. All samples had RIN (RNA integrity number) values of 9.8 or higher (scale from 1 to 10). Technical replicates were segregated onto separate array slides to control for slide-to-slide staining variation. Staining quality control was assessed using GenomeStudio (Illumina). Next, evaluation of variation across technical replicates was performed using principal component analysis, which showed the C4-2 array data were unreliable and they were not used in the study. Array data were analyzed using the limma package for the R statistical program (Ritchie et al., 2015). Arrays were background corrected, and normalized and gene-wise linear model fits were calculated. An empirical Bayes framework was used to evaluate gene variance to reduce false positives and increase power and significance. Moderated F-statistics that aggregated the t-statistics for all the compared technical replicate sets were used to create an overall significance measure for each gene.

### 2.6 Invasion and migration assays

FAC-sorted cells were plated in serum-free media 8 µm pore transwells (FALCON #353097) in 24-well plates (FALCON #353504), with 200,000 cells per well coated in Matrigel (invasion) or collagen type I (migration), and then, 18 h (migration) or 12 h (invasion) was given to invade across the membrane; 10% FBS media was used as the attractant. The transwells were fixed with 4% paraformaldehyde washed and stained with .12 mg/mL of crystal violet dye (Becton Dickinson #212526), dried, and then, imaged using a Keyence bz-x800 automated microscope.

### 2.7 Growth curve

FAC-sorted cells were plated in 96-well plates (CELLSTAR #655180), 1,000 cells per well, and read at 24, 48, 72, 96, 120, and 144 h time points using 10-µL CCK-8 reagent (Dojindo) per well, after a 3 h incubation read using a spectrophotometer at 490 nm.

### 2.8 Anoikis resistance

FAC-sorted cells were plated in 96-well plates coated in PolyHEMA (Sigma #P3932), and then, the cell viability of 8,000 cells per well was determined 48 h later using 10 µL CCK-8 reagent (Dojindo) per well; after 3 h of incubation, a spectrophotometer at 490 nm was used to read.

### 2.9 Luciferase assays

FAC-sorted cells were placed in 24-well plates (FALCON #353504), 200,000 cells per well, and 24 h later, they were transfected using the JetPRIME transfection reagent (Polyplus). Subsequently, 48 h later, media ertr collected and frozen, and then, aliquots of media were analyzed using the Secrete-Pair™ dual luminescence assay kit (GeneCopoeia #LF033), and luminescence was read using a CLARIOstar (BMG Labtech) microplate reader. Gaussia luciferase was read at 480 nm, and transfections were normalized by reading secreted alkaline phosphatase at 405 nm; Welch’s *t*-test was used to test significance.

### 2.10 3’ RACE PCR

Total mRNA was extracted using the RNeasy mini kit (Qiagen #74104) on a column. DNase digestion was performed (Qiagen #79524), and then, the resultant mRNA was used with the system for rapid amplification of cDNA ends (3’ RACE) kit (Invitrogen #18373019).

### 2.11 Western blot analysis

Cell lysates were collected in the RIPA buffer, and Western blots were carried out using PVDF membranes (Millipore). After blocking, the membranes were incubated with appropriate dilutions of specific primary antibodies (1:250 dilution of anti-ONECUT2 and 1:2,500 dilution of anti-GAPDH (HRP-conjugated) antibodies) overnight at 4°C.

### 2.12 Identification of OC2-correlated genes in prostate cancer

To identify OC2-correlated genes, we collected six transcriptome data form the prostate cancer patients (Supplementary Figure S2A). For each dataset, we calculate Spearman’s rho and *p*-value between OC2 and all genes. Next, the genes with Spearman’s rho more than 0.25 and *p*-value lower than 0.05 were selected as potential OC2-correlated genes. Among them, the genes that were detected as OC2 highly correlated genes from four or more datasets were defined as OC2-correlated genes. The combined correlation score for each gene was calculated using the following equation:

$${CCS}_{i}=\frac{\sum_{n=1}^{N_{i}}R_{i}}{\sqrt{N_{i}}},$$

where *CCS*_*i*_ is the combined correlation score of the *i*th gene, N_*i*_ is the number of datasets that were detected as OC2 highly correlated genes of the *i*th gene, and R_*i*_ is Spearman’s correlation rho of *i*th genes and OC2. The correlation score summarizes the Spearman’s rho of each dataset and reflects the number of the detected datasets.

### 2.13 MiRNA target interactome and binding sites

The miRNA target interaction information was downloaded from the TargetScan database. The TargetScan database provides a ‘summary counts and default predictions’ file that has the predicted miRNA target interaction with information on the number of binding sites. To achieve reliable miRNA target interactome, we selected miRNA target interactions that were predicted at least once using three other miRNA-target prediction tools, namely, miRDB, TarBase, and MicroRNA.org (miRDB: PMID: 30670076, TarBase: PMID: 16373484, and MicroRNA.org: PMID: 18158296).

### 2.14 Enrichment test of OC2-correlated genes for miRNA targets

We performed the enrichment test of OC2-correlated genes for each miRNA target. We counted overlapped genes of OC2-correlated genes and each miRNA target, and Fisher’s exact test was used to assess enrichment of the miRNA targets.

### 2.15 Reconstruction of the OC2–miRNA target network

Among the 43 miRNAs that can bind to OC2, we selected the top five significantly enriched miRNAs that have a large number of OC2-correlated genes as a target. These five miRNAs, their OC2-correlated target genes, and OC2 were used as nodes, and information on the number of binding sites was used as edges in the network. Next, we performed functional enrichment tests on the network genes using DAVID software. We discard nodes that do not belong to the major BP. The nodes are grouped and arranged as given in the enriched biological process. We colored nodes and edges according to the combined correlation score and the number of binding sites, respectively. The node size represents the length of 3’ UTR.

### 2.16 Statistical analysis

Cox proportional hazard regression analysis was used to calculate the hazard ratio and *p*-value. The Kaplan–Meier plot was used to present the cumulative hazard function.

## 3 Results

### 3.1 ONECUT2 mRNA contains a long 3’ UTR

Previously, Rotinen et al. (2018) and Guo et al. (2019) showed that the OC2 transcription factor is a master regulator that drives aggressive prostate tumors and androgen insensitivity and activates a neuroendocrine differentiation network. A review of ONECUT2 in the context of neuronal differentiation and development indicated that the transcription factors ONECUT1 (OC1) and ONECUT2 (OC2) function coordinately in neural development (Goetz et al., 2014; Klimova et al., 2015; Delile et al., 2019; van der Raadt et al., 2019).

**FIGURE 1:**
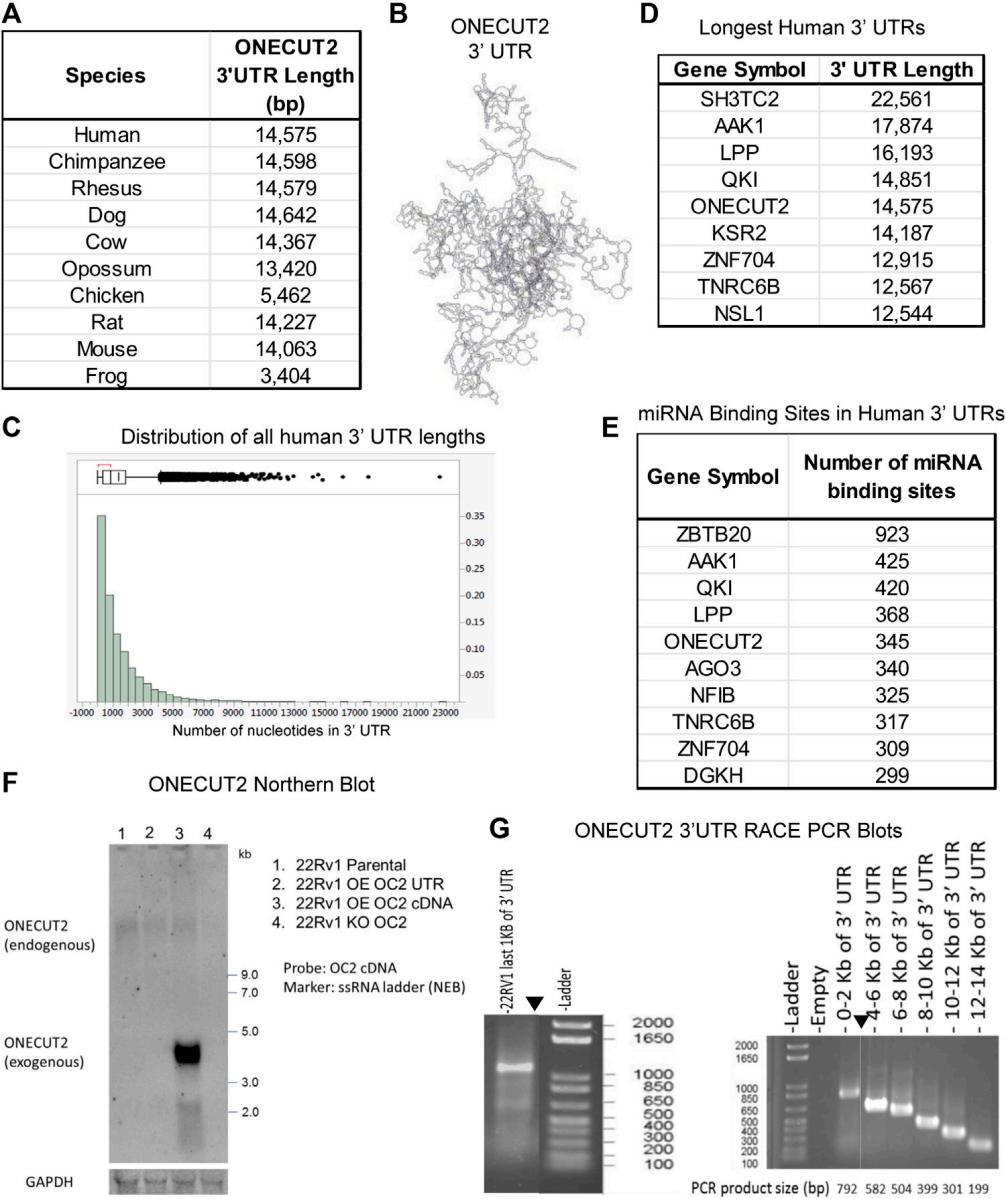
ONECUT2 mRNA contains a long 3′ UTR. **(A)** Table of lengths OC2 3′ UTR (TargetScan 6.0). **(B)** Model of 3′ UTR structures of OC2. **(C)** Histogram of 3′ UTR lengths in the human genome. **(D)** Table of genes with the longest 3′ UTRs in the human genome. **(E)** Table of genes with the most miRNA-binding sites in the human genome. **(F)** Autoradiograph from Northern blot showing the full-length OC2 mRNA was present in all 22Rv1 cells but lowered in the OC2 knockout. **(G)** Left: Gel capture of the nested 3′ RACE PCR product of the last 1,000 base pairs of OC2 ‘3 UTR. **(G)** Right: Gel capture of the nested 3′ RACE-walking PCR products along the length of OC2 ‘3 UTR.

*ONECUT2* has a very long 3′ UTR (14,757 nt). OC2 mRNA contains two exons, starting with a 343 nt 5′ UTR and a 1,228 nt coding sequence, interrupted by a 34,492 nt intron trailed by a short 287 base pair coding sequence, and, last, a 14,575 nt 3′ UTR. OC2 3′ UTR is very long in most species (Figure 1A). This extreme length of 3‘ UTR creates a complex macromolecule as illustrated in Figure 1B. Additionally, the OC2 3’ UTR sequence is highly conserved (Supplementary Figures S1A, B). This conservation in both size and sequence points to OC2 3′ UTR playing an important role that has been subjected to evolutionary pressure to maintain this length.

Because of the extreme length of the OC2 transcript, there was concern that OC2 3′ UTR might undergo alternative translation within 3′ UTR to produce novel proteins, as discussed (Lee et al., 2018; Ma L et al., 2019; Ji et al., 2015). We analyzed 3’ UTR in all three frames to look for potential proteins using EMBOSS six pack software (Chojnacki et al., 2017) and eight potential frames were identified, but these possible peptide sequences could not be identified in human proteomic data using the peptide atlas (Desiere et al., 2006; Deutsch et al., 2015).

Using TargetScan6 data (Lewis et al., 2005), 3′ UTRs were sorted by the longest variant of each gene transcript. A histogram of these UTR lengths is seen in Figure 1C. This analysis placed OC2 3′ UTR as the fifth longest 3′ UTR in the human genome, more than 17 times longer than the median 3′ UTR length. The top eight longest UTRs are shown (Figure 1D). To further confirm the length of OC2 3’ UTR, we used the TREGT (top-ranked transcript isoforms in human protein-coding genes) web resource (Tung et al., 2020) to assess alternate OC2 transcripts, and the long transcript is the predominant transcript in 98.30% of the samples (over 17,000 RNAseq runs consisting of 54 different tissue types from 948 donors).

Because of the importance of 3′ UTRs in regulation of translation, we determined the total number of miRNA-binding sites or microRNA response elements (MREs) across all human genes. OC2 has the fifth most MREs in the human genome (Figure 1E). Next, the MRE density was calculated for all genes by dividing the number of nt by the total number of microRNA-binding sites present. The OC2 MRE density was equivalent to the median MRE density of all genes. It is to be noted that OC2 mRNA contains 13 conserved miR-9 MREs and another 13 non-conserved miR-9 MREs, which pointed to a hypersensitivity to miR-9 binding and RISC-mediated degradation, as noted previously by Plaisance et al. (2006); Zhang et al. (2015); Delaloy et al. (2010); Shen et al. (2019). Northern blot analysis confirmed that full-length OC2 mRNA is present in 22Rv1 prostate cancer cells (Figure 1F). 3′ RACE PCR, using nested primers, was performed following DNAse treatment. The first RACE reaction only tested the last 1,000 nt of UTR, while the second RACE PCR walked from the end of UTR throughout the coding sequence (Figure 1G left and right). Taken together, OC2 mRNA has an exceptionally long 3’ UTR that is conserved, complex, and contains many microRNA-binding sites.

### 3.2 ONECUT2 mRNA is potentially a ceRNA

The size of OC2 3′ UTR and the number of MREs it contains suggested that it might be an ideal competitive endogenous RNA (ceRNA) as was speculated by Sarver & Subramanian (2012) in their creation of the first ceRNA database. ceRNAs are mRNAs that post-transcriptionally regulate each other by competing for their mutually binding miRNAs. The two transcripts compete to sponge miRNAs away from each other and relieve miRNA-mediated repression. Three separate *in silico* ceRNA network analyses were undertaken to establish what an OC2-driven ceRNA network would consist of and what genes and miRNA partners such a network would modulate. The first network used TCGA prostate cancer data, along with data from the work of Taylor et al. (2010), to determine genes that are both strongly positively correlated (greater than 0.5 Spearman correlation) and with at least five shared MREs between the gene and OC2. To further filter these genes, we used a second dataset that modeled the androgen-insensitive transformation of tumor cells undergoing androgen therapy (D’Antonio et al., 2008). Because this dataset was relatively limited in number, we increased the correlation threshold to 0.65 (Spearman correlation). Gene Ontology analysis of these ceRNA partner genes indicated that neurogenesis was the top biological category. One weakness of this initial approach, however, is that the majority of tumors in these datasets are of the more typical adenocarcinoma subtype, whereas CRPC tumors are of multiple subtypes (You et al., 2016), meaning that many of these tumors did not employ OC2 as a significant cancer driver.

To overcome this limitation, a second network was developed. The TCGA and Taylor dataset tumors were segregated into quartiles according to the expression of OC2. The top and bottom quartiles were used exclusively to represent OC2-driven tumors and tumors driven by OC2-independent mechanisms, respectively. As previously noted by Rotinen et al. (2018), neuroblastoma (NB) and small-cell lung cancers (SCLCs) express high levels of OC2 mRNA. Gene sets derived from these two cancers (Sato et al., 2013; van Nes et al., 2013) were likewise quartiled by OC2 expression. The resulting expression profiles were queried for genes with more than five shared MREs and high positive correlation with OC2 expression. Again, Gene Ontology indicated that these ceRNA partners were significantly involved in neurogenesis and neuronal activities.

This list of ceRNA partners was then analyzed to identify which miRNAs were the most prevalent ceRNA–miRNA–ceRNA effectors. The top thirty most prevalent miRNAs were compared to their expression in prostate cancer, NB, and SCLC. miRNAs were then filtered based on expression in target tissues; those remaining were miR-9, miR-124, miR-27, miR-29, miR-30, and miR-181. Because miR-9 MREs are the most abundant on OC2 3′UTR, this result implied that OC2 was sponging miR-9. Studies in neurons had previously shown that short 3′ UTR OC1 could be rescued by the mir-9 sponge to induce chromatin remodeling (Luxenhofer et al., 2014; van der Raadt et al., 2019). To date, the cancer literature suggests OC2 is sensitive to miR-9 targeting (Plaisance et al., 2006; Zhang et al., 2015; Shen et al., 2019). Moreover, miR-124 and miR-9 both target neurogenic genes and function together as transcriptional modulators in neuronal development via EZH2 modulation (Abernathy et al., 2017; Lee et al., 2018).

**FIGURE 2:**
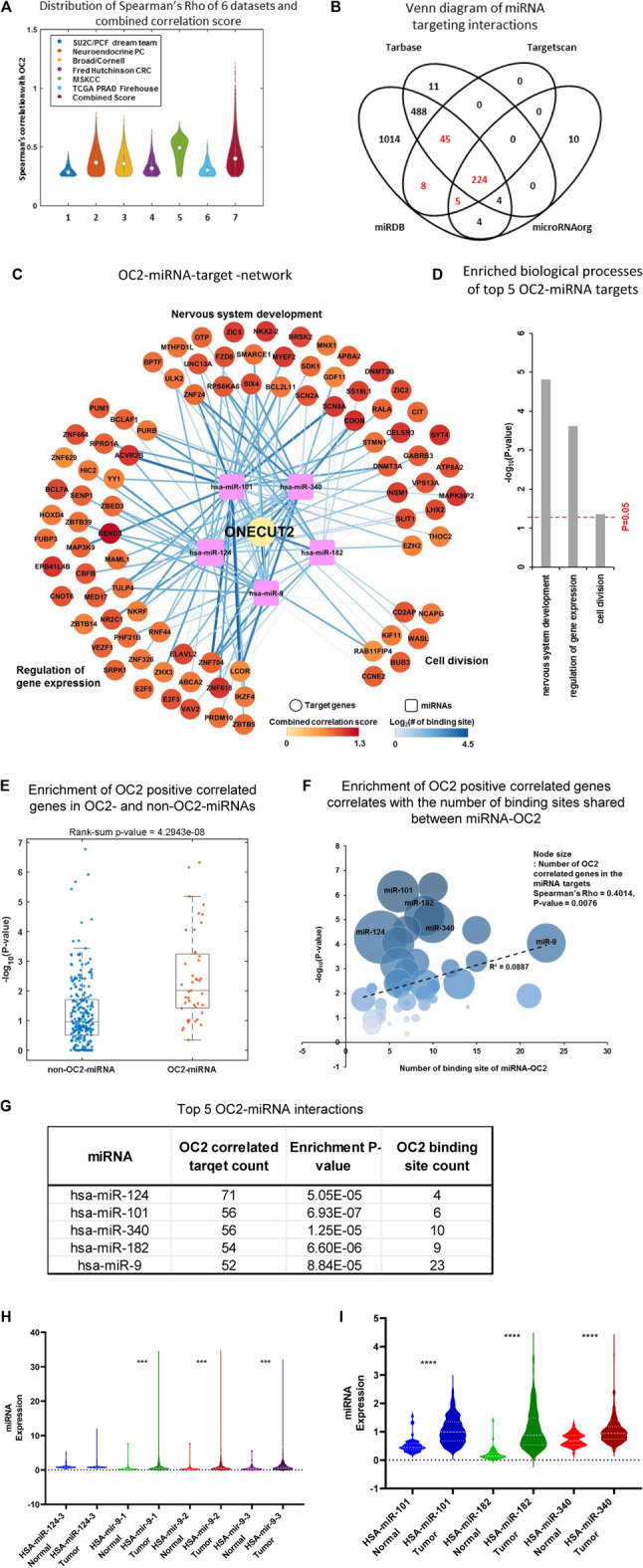
ONECUT2 mRNA is potentially a ceRNA. **(A)** Distribution of Spearman’s rho of OC2 highly correlated genes from six datasets and their combined score. **(B)** Comparison of multiple public miRNA-targeting databases lists of miRNAs that are predicted to bind to OC2 3′ UTR. **(C)** OC2–miRNA target network. The circular nodes are OC2 highly correlated genes that have miRNA-binding sites. The rectangle nodes are top five miRNAs that can bind to OC2. The node size represents the length of 3′ UTR. The node color represents the combined score. Edges show the miRNA target-binding information. The color and width of the edge represent the number of binding sites. **(D)** Gene Ontology of the OC2–ceRNA network. **(E)** Enrichment of OC2 highly correlated genes in the targets of OC2 targetable and non-targetable miRNAs. **(F)** Bubble plot showing the enrichment of OC2 highly correlated genes and the number of binding sites shared between miRNA and OC2. **(G)** Table of miRNA–ceRNAs interactors ordered by the number of targets. **(H)** Violin plots of miRNA expression in tumors vs. normal tissue higher overall miRNA expression. **(I)** Violin plots of miRNA expression in tumors vs. normal tissue lower overall miRNA expression.

A final OC2 ceRNA network was developed using six large, publicly available prostate cancer datasets derived from 1,175 tumors (Abida et al., 2019; Beltran et al., 2016; Barbieri et al., 2012; Kumar et al., 2016; Taylor et al., 2010); see Supplementary Figure S2A for dataset overview. For each dataset, we calculated Spearman’s rho and *p*-value between OC2 and all genes. Next, the genes with Spearman’s rho >0.25 and *p*-value <0.05 were selected as potential OC2-correlated genes. Among these, the genes identified as OC2 highly correlated genes from four or more datasets were defined as OC2-correlated genes (Figure 2A). The correlation score summarizes the Spearman’s rho of each dataset and reflects the number of the total number of datasets with high OC2 correlation (Supplementary Figure S2B). We combined four OC2-miRNA databases to find miRNAs that were predicted to bind to the OC2 mRNA in at least three out of the four (Figure 2B).

The final ceRNA network prediction is shown in Figure 2C (total network shown in Supplementary Figure S2C). The five most significant miRNAs generated three nodes whose gene ontologies were 1) nervous system development, 2) regulation of gene expression, and 3) cell division (Figure 2D). Genes positively correlated with OC2 were segregated into those that shared miRNAs with OC2 and those without any shared miRNAs, and the genes with shared miRNAs were significantly enriched and displayed a ceRNA effect (Figure 2E). Likewise, the genes that have more shared MREs are enriched for more significant positive correlation (Figure 2F). The top five miRNAs are shown in Figure 2G. The expression of these five miRNAs in prostate tissue and cancer was assessed using TCGA tumor and non-cancerous tissue RNAseq data (Figures 2H, I). All miRNAs had significantly higher expression in tumor tissue than normal prostate tissue, with the exception of miR-124, which was increased but did not meet the significance threshold.

The complex OC2 UTR structure seen in Supplementary Figure S2E shows the 13 highly conserved miR-9-binding sites. We postulated that this bulky structure could allow miRNAs to bind but would block the RNA-induced silencing complex (RISC) (Hammond et al., 2000) proteins from having access to the miRNA–mRNA complex. The individual hub of miR-9–ceRNA interactions is shown in Supplementary Figure S2F.

### 3.3 ONECUT2 3’ UTR is resilient to miRNA targeting

**FIGURE 3:**
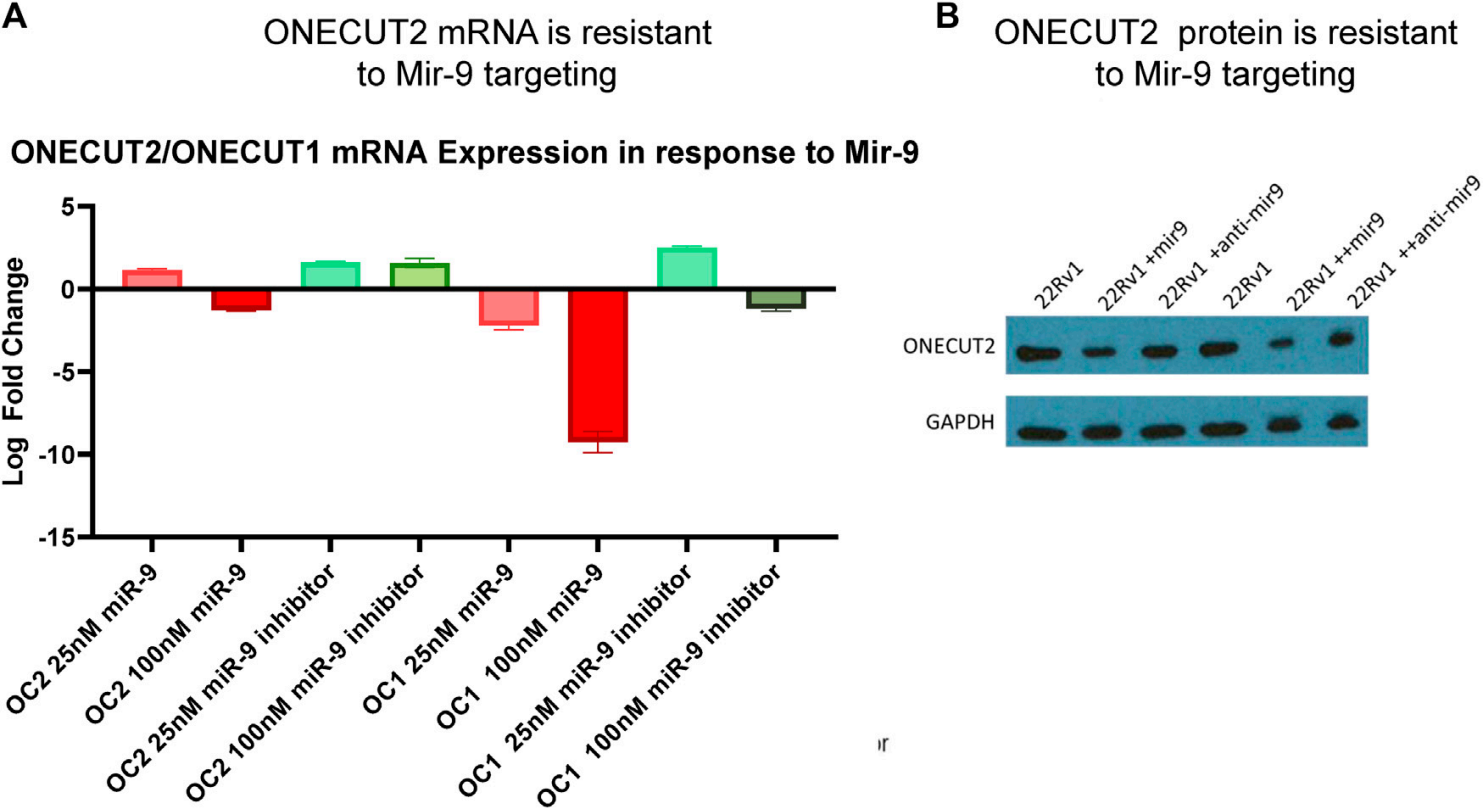
ONECUT2 3′ UTR is resilient to miRNA targeting. **(A)** Real-time PCR data from 22Rv1 cells, OC1, and OC2 mRNA after treatment with miR-9 and miR-9 inhibitor showing that OC2 was insensitive to miR-9. **(B)** Capture of the autoradiograph from Western blot showing the OC2 protein after treatment with miR-9 and miR-9 inhibitor showing OC2 was insensitive to miR-9.

In order for OC2 to be an effective ceRNA, 3′ UTR must be expressed in full length and be resilient to miRNA-targeted degradation. Because several studies (Plaisance et al., 2006; Zhang et al., 2015; Shen et al., 2019) reported that OC2 was targeted by miR-9 and sensitive to RISC machinery, we tested OC2 resilience to miRNA degradation. It is important to note that in these publications, OC2 UTR reporters were not tested using the full-length 3’ UTR of OC2, but rather fragments ranging from 800 to 3000 base pairs, ensuring that the highly complex mRNA structure would not be present and could be more easily targeted by RISC. Twelve hours after miRNA transfection, OC2 expression was only minimally changed, even when exposed to high-dose 100 nM oligo (Figure 3A). Likewise, loss of the OC2 protein was only seen after multi-day dosing of 100 nm of miR-9 (Figure 3B).

### 3.4 ONECUT2 protein and 3’ UTR drive analogous networks

The likelihood for OC2 to be a potent ceRNA from the *in silico* network data warranted experimental testing. However, the length of OC2 3′ UTR precluded the use of typical viral infection because of the inefficiency of packing such a long vector inside a viral particle. We used a continually selected vector that had the GFP sequence flanked by the entire sequence of OC2 3’ UTR (Supplementary Figure S4A, left).

**FIGURE 4:**
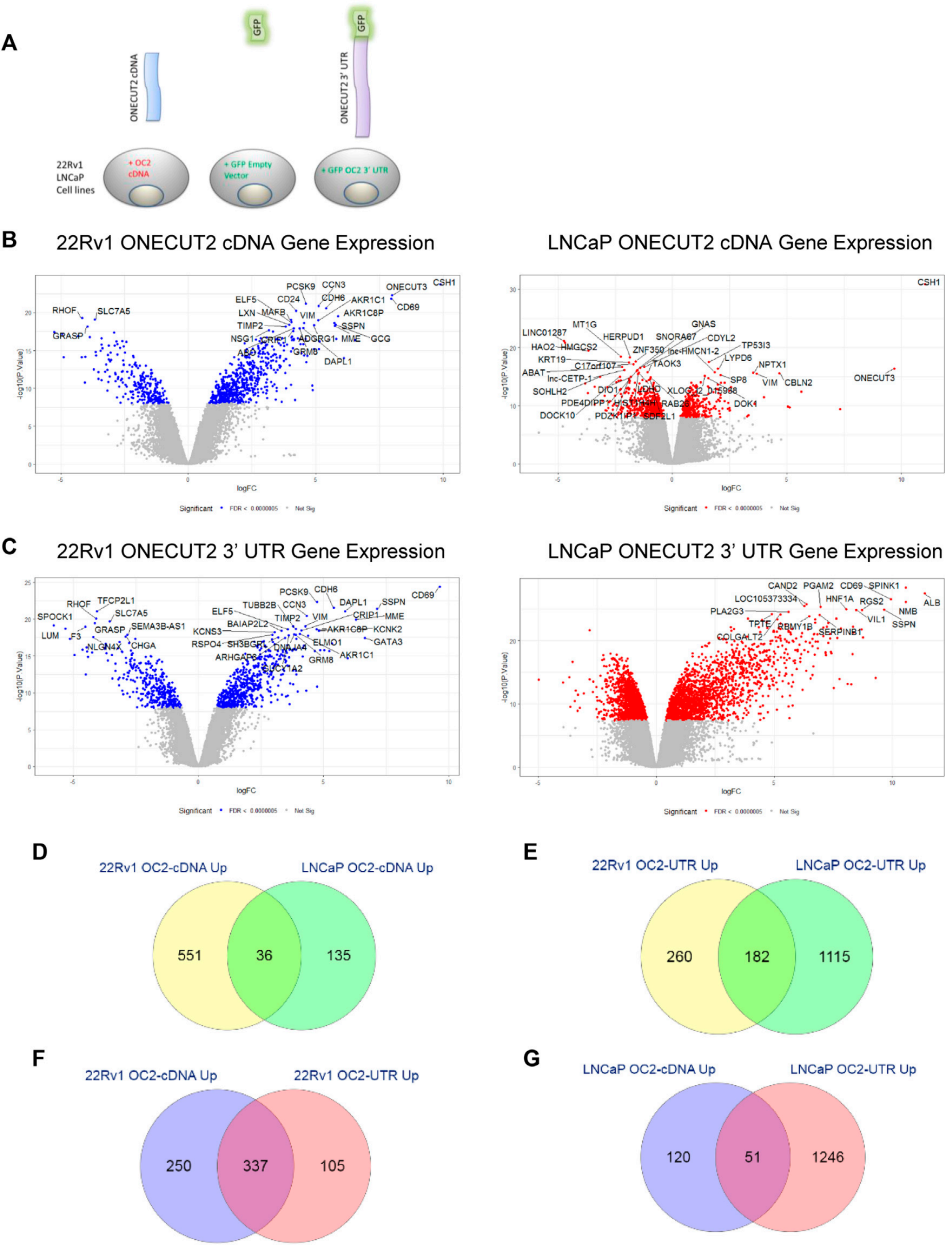
ONECUT2 protein and 3′ UTR drive analogous networks. **(A)** Schematic of the vector products used to create the LNCaP, C4-2, and 22Rv1 prostate cancer lines. **(B)** Left and right: Volcano plots of significantly differentially expressed genes in OC2 cDNA overexpressing cells 22Rv1 (left) and LNCaP (right) (significance = FDR <0.0000005). **(C)** Left and right: Volcano plots of significantly differentially expressed genes in OC2 3′ UTR overexpressing cells 22Rv1 (left) and LNCaP (right) (significance = FDR <0.0000005). **(D)** Overlap of genes significantly overexpressed by OC2 proteins between 22Rv1 and LNCaP. **(E)** Overlap of genes significantly overexpressed by OC2 3′ UTRs between 22Rv1 and LNCaP. **(F)** Overlap of genes significantly overexpressed between OC2 protein and 3′ UTR in 22Rv1. **(G)** Overlap of genes significantly overexpressed between OC2 protein and 3′ UTR in LNCaP. **(M)** ChIP-X enrichment analysis (ChEA) and ENCODE transcription factor ChIP-seq databases analysis to determine downstream transcription factors: 22Rv1 OC2 protein (green), 22Rv1 OC2 3′ UTR (blue), LNCaP OC2 protein (red), and LNCaP OC2 3′ UTR (orange). **(N)** A four-way Venn diagram of all LNCaP OC2 cDNA/3′UTR and 22Rv1 OC2 cDNA/3′UTR significantly upregulated genes. **(O)** Left: The overlap of significantly enriched Hallmarks and KEGG canonical pathways for 22Rv1 OC2 protein/OC2 3′UTR. Right: The overlap of significantly enriched Hallmarks and KEGG canonical pathways for LNCaP OC2 protein/OC2 3′UTR. **(P)** 1, 2, 3, and 4: Gene set enrichment analysis of OC2 protein and 3′ UTR show similar significant PRC activity. **(Q)** 1, 2, 3, and 4: Gene set enrichment analysis of OC2 protein and 3′ UTR show similar significant PRC activity. **(R)** 1) Gene set enrichment analysis of OC2 protein and 3′ UTR show that OC2 3 ‘UTR has increased transcription factor E2F activity. 2) OC2 3’ UTR has significantly cholesterol homeostasis activity. 3) OC2 3′ UTR shows significantly increased activity of MYC. 4) OC2 protein-induced genes are enriched for p53-signaling activity that is not seen in the OC2 3′ UTR-induced genes.

Additionally, cells were created that overexpressed the OC2 cDNA protein alone to determine the effect of the OC2 transcription factor without 3′ UTR (Supplementary Figure S4A, right). These constructs were each created in the LNCaP and 22Rv1 prostate cancer lines as seen in Figure 4A. Supplementary Figures S4B, C shows respective GFP and mCherry cell positivity compared to parent cell autofluorescence. Since all three of the OC2 3’ UTR ceRNA *in silico* networks implicated both miR-9, cells were created that overexpressed miR-9 along with an mCherry reporter.

The gene networks created by the overexpression of the empty GFP vector, OC2 cDNA, OC2 3’ UTR, and miR-9 were assessed by Agilent 60K microarray. Evaluation of variation across technical replicates was performed using principal component analysis. Array data were analyzed using the limma package for the R statistical program (Ritchie et al., 2015).

Differential expression values were then obtained and plotted as volcano graphs (Figure 4B, left and right). OC2 cDNA overexpression had a lower effect on gene expression in the LNCaP cell background than the 22Rv1 cell background, but still indicated overlap of overexpressed genes between LNCaP and 22Rv1 (Figure 4D). The number of genes significantly overexpressed due to enforced OC2 3′ UTR was surprising, as was the strength of the overexpression (Figure 4C, left and right panels). The two sublines also had abundant overlap of their overexpressed genes (Figure 4F). The OC2 UTR and cDNA-induced genes were highly similar; 78% of the OC2 3′ UTR overexpressed genes also overexpressed in OC2 cDNA cells (Figure 4E and right panels of Figures 4B, C). This overlap of OC2 3′ UTR and OC2 cDNA upregulated genes was weaker in LNCaP; only 29% of OC2 3′UTR overexpressed genes were also overexpressed in OC2 cDNA (Figure 4G). The overexpression of miR-9 caused many more genes to be under-expressed rather than overexpressed, which was unsurprising, as microRNAs typically target mRNA transcripts for degradation (Supplementary Figure S4E).

The significantly modulated genes were then characterized using the Enrichr database (Chen et al., 2013; Kuleshov et al., 2016)*.* Gene Ontology analysis showed the OC2 3′UTR and cDNA networks in both cell sets had signatures implicating developing neurons (Supplementary Figures S4F, G). The **a**ll RNA-seq and ChIP-seq sample and signature search (ARCHS^4^) (Lachmann et al., 2018) server was used to analyze the most significantly changed gene expression from the microarray data. The OC2 networks implicated numerous highly significant transcription factors (Supplementary Figures S4H, I). In addition, OC2 was confirmed as a significant implicated transcription factor (bolded in S4H and S4I).

The OC2-driven networks were analyzed to determine downstream transcription factors using ChIP-X enrichment analysis (ChEA) and ENCODE transcription factor ChIP-seq databases (Kuleshov et al., 2016) (Supplementary Figure S4J). This analysis implicated polycomb-repressive complex (PRC) members *SUZ12* and *EZH2*, as well as the pluripotency driving transcription factors *SOX2* and *NANOG*. Additional transcription factors implicated were RE1-silencing transcription factor (*REST*), previously known as neuron-restrictive silencer factor (NRSF), known to repress neuronal genes in non-neuronal cells and interact with androgen receptor. The OC2 networks were then assessed to see which microRNAs exhibited the most significant change in their targets (Supplementary Figure S4K). This indicated that miR-124 and miR-9 were the 3rd and 12th highest ranked miRNAs by target enrichment. A four-way Venn diagram of all OC2 cDNA/UTR significantly upregulated genes is seen in (Supplementary Figure S4L). The four-way union consisted of just 12 genes: *ADGRF1*, *CBLN2*, *CCN3*, *CLU*, *CRABP1*, *CXCR4*, *IFITM1*, *MDK*, *NLGN1*, *STOM*, *SYT4*, and *TSPAN7.* Four of these genes are involved in neurobiology: *CBLN2* (cerebellin 2 precursor), *MDK* (Midkine previously known as neurite outgrowth-promoting factor 2), *NLGN1* (neuroligin 1), and *SYT4* (synaptotagmin 4). Target analysis of miRNA showed the top predicted miRNA by *p*-value (*p* = 0.001741) was miR-9. Because the low number of upregulated genes in the LNCaP OC2 cDNA cells diminished the four-way union to just 12 genes, we added the 128 genes from the three-way union of LNCaP OC2 3′ UTR +22Rv1 OC2 3’ UTR + 2Rv1 OC2 cDNA to the previous four-way union twelve genes and performed gene ontology analysis using Enrichr. This indicated SUZ12 was the top implicated transcription factor. The top significant biological processes were all metabolic processing of compounds, likely due to the upregulation of the Aldo-Keto reductase family members (*AKR1C1*, *AKR1C3*, *AKR1C4*, and *AKR1C8P*). The biological process synaptic assembly was significantly associated with OC2-induced genes. The cellular components most significantly implicated by OC2 overexpressed genes were dendrites and focal adhesions.

Gene set enrichment analysis (GSEA) was performed using the UCSD/broad software (Mootha et al., 2003; Subramanian et al., 2005)*.* We validated the OC2 network against the previous OC2 upregulated gene signature developed by Rotinen et al. (2018). The result showed extremely high concordance with this previous OC2-driven network (Supplementary Figures S4M1, M2). We took a top-down approach to GSEA, starting with the broader Hallmarks and KEGG canonical pathways signatures and then using increasingly more specific signature sets such as oncogenic and later prostate cancer specific signatures. The overlap of signatures was numerous for the Hallmarks and KEGG canonical pathways (Supplementary Figure S4N, right and left) (Supplementary Figure S4P). Among these highly enriched overlapping signatures were both epithelial mesenchymal transition and neuroactive ligand receptor interaction signatures (Supplementary Figures S4O1, O2). The direct differences between the two OC2 gene networks were that the OC2 3’ UTR drove significantly higher enrichment for E2F activity, cholesterol homeostasis, and MYC targets, while OC2 cDNA gens had enriched p53 signaling (Supplementary Figure S4Q panels 1, 2, 3 and 4). Oncogenic specific GSEA signatures showed enrichment for EZH2 activity for both OC2 networks. Prostate cancer-specific tested GSEAs showed significant enrichment for metastasis (Supplementary Figures S4R1, 2) and hypermethylation signatures (Supplementary Figures S4S1, 2S). The high gene set enrichment scores of the OC2 networks for hypermethylation and EZH2 suggests increased PRC activity. As a result, a set of PRC-linked signatures was compiled and then tested, and both the OC2 networks had significant enrichment for PRC selected signatures shown in Supplementary Figures S4T1–4.

From this gene expression analysis, we can conclude that this OC2 cDNA network is analogous to the OC2 data previously described by Rotinen et al. (2018). To this, we add OC2 3′ UTR and protein network-driven neuronal developmental processes and oncogenic processes such as metastasis and epithelial-to-mesenchymal transition and activate PRC activity. Most importantly, these data showed that 3’ UTR is capable of regulating gene expression and activating oncogenic networks without concomitant expression of the OC2 protein.

### 3.5 ONECUT2 protein and 3’ UTR mRNA independently increase PRC activity

**FIGURE 5:**
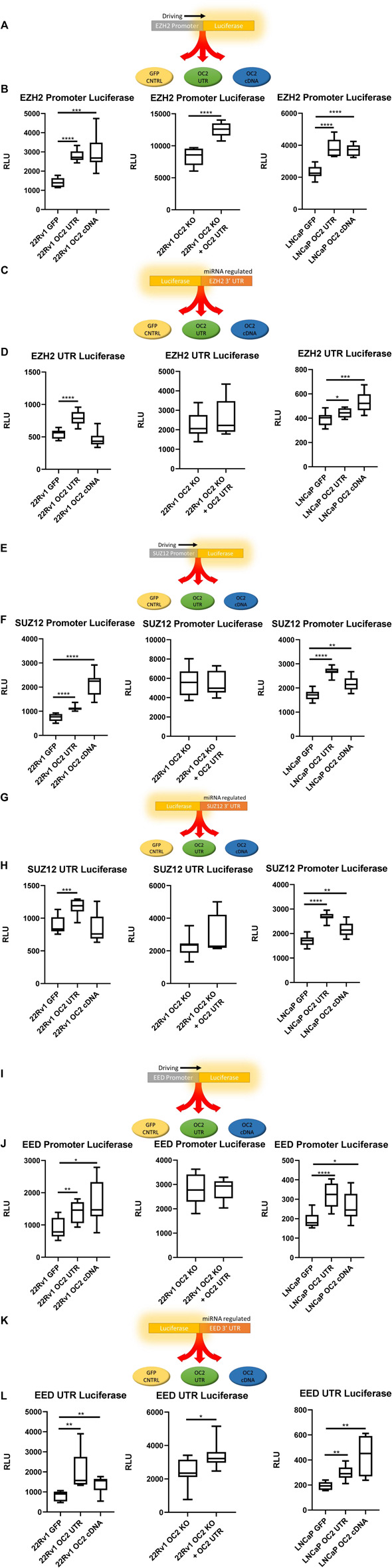
OC2 Protein and mRNA coordinate to increase PRC activity **(A)** EZH2 Promoter reporter activity schematic. **(B)** EZH2 Promoter luciferase activity graphs, 22Rv1 sublines (left) 22Rv1 OC2 Knock Out (center) and LNCaP sublines (right). **(C)** EZH2 3′ UTR reporter activity schematic. **(D)** EZH2 3′ UTR luciferase activity graphs, 22Rv1 sublines (Left) 22Rv1 OC2 Knock Out (Center) and LNCaP sublines (Right). **(E)** SUZ12 Promoter reporter activity schematic. **(F)** SUZ12 Promoter luciferase activity graphs, 22Rv1 sublines (Left) 22Rv1 OC2 Knock Out (Center) and LNCaP (Right). **(G)** SUZ12 3′ UTR reporter activity schematic. **(H)** SUZ12 3′ UTR luciferase activity graphs, 22RV1 (Left) 22Rv1 OC2 Knock Out (Center) and LNCaP sublines (Right). **(I)** EED Promoter reporter activity schematic. **(J)** EED Promoter luciferase activity graphs, 22Rv1 sublines (Left) 22Rv1 Onecut2 Knock Out (Center) and LNCaP (Right). **(K)** EED 3′ UTR reporter activity schematic. **(L)** EED 3′ UTR luciferase activity graphs, 22RV1 (Left) 22Rv1 Onecut2 Knock Out (Center) and LNCaP sublines (Right).(Significance determined using Welch’s T-test *p*-Values *<.05, **<.005, ***<.0005, ****<.00005).

To confirm the EZH2 and PRC gene set enrichment findings from the microarray analysis, we used luciferase activity assays to assess the ability of OC2 protein and 3′ UTR to modulate PRC activity *in vitro* (Figure 5A). Two different dual-luciferase systems were employed, one using the promoters of PRC to test for transcription activity changes and a second set to test the modulation of the translational activity using the 3’ UTRs of PRC genes (Supplementary Figures S5A, SF5B).

The OC2 3′ UTR and OC2 protein were shown to be capable of inducing significant *EZH2* transcriptional activity, via promoter reporter in both 22Rv1 and LNCaP backgrounds (Figure 5B, left and right). We confirmed if this OC2 3′ UTR-driven increase of *EZH2* transcription was independent of OC2 transcription factor activity by using 22Rv1 cells with CRISPR-engineered knockout of OC2 that was then transfected with the OC2 3’ UTR (Supplementary Figure S5C). This experiment showed that the increase of *EZH2* transcription was also independent of OC2 protein (Figure 5B, center).

The ability of OC2 to influence EZH2 translation was tested by transfection with the *EZH2* 3′ UTR regulating the GLuc/SEAP luciferase mRNA as seen in Figure 5C. In the 22Rv1 cell line, the overexpression of the OC2 3′ UTR increased luciferase when controlled by the EZH2 3′ UTR, whereas OC2 protein did not significantly change the activity (Figure 5D Left). When OC2 protein was knocked out and the 3′ UTR overexpressed, the EZH2 translational activity was increased but highly variable. This variability precluded the difference from reaching statistical significance (Figure 5D, center). The LNCaP cell line background showed an increase of EZH2 translational signal in response to the overexpression of both the OC2 3’ UTR and the OC2 protein (Figure 5D, right), demonstrating that the mechanisms of modulation are not solely through ceRNA sponging.

We assessed whether *SUZ12* activity was sensitive to OC2 modulation using the GLuc/SEAP system (Figures 5E, G). In both 22Rv1 and LNCaP backgrounds, the OC2 3′ UTRs and the OC2 proteins increased *SUZ12* promoter activity (Figure 5F, left and right). The increases were inconsistent between the two cell types. However, the knockout of OC2 protein abolished the increase of *SUZ12* promoter activity due to OC2 3’ UTR overexpression, suggesting that the increase maybe be dependent on upstream OC2 transcription factor activity (Figure 5F, center).

Regulation of *SUZ12* mRNA by OC2 protein or 3′ UTR was not seen in the LNCaP sublines (see Figure 5H, right). In 22Rv1 cells, only the overexpression of OC2 3′ UTR was capable of significantly increasing SUZ12 3′ UTR activity (Figure 5H, left). The effects of the OC2 3’ UTR to modify SUZ12 activity in an OC2 cDNA knockout cell was not significant (Figure 5H, center).

A third member of PRC, Embryonic Ectoderm development protein (EED), was assessed for transcriptional/translational susceptibility to OC2 changes. The same dual-luciferase system was used (Figures 5I, K). Both OC2 protein and 3′ UTR were able to increase EED promoter activity (Figure 5J, left and right). Loss of the OC2 protein negated any impact from the overexpression of OC2 3′ UTR (Figure 5J, center) indicating that the increase of transcriptional activity due to the overexpression of the OC2 3’ UTR is dependent on functional OC2 protein.

The EED mRNA 3′ UTR was particularly sensitive to OC2 increases (Figure 5L, left and right). In 22Rv1, the OC2 protein and 3′ UTR increased luciferase activity significantly and similarly in LNCaP cells. Knocking out the OC2 protein diminished but did not abrogate the significant increase of EED 3′UTR regulated luciferase activity due to overexpression on the OC2 3′ UTR (Figure 5L, center). EED is a downstream target of OC2 by promoter activity due to the fact that both OC2 protein and its mRNA are positively regulated by increases in both the OC2 protein and the OC2 mRNA.

Our *in silico* network and microarray analyses implicated EZH2 and PRC as being induced by both OC2 ceRNA and the OC2 transcription factor. Using luciferase assays, we confirmed this in two prostate cancer backgrounds. The OC2 knockout luciferase data showed that the OC2 3′ UTR can work either independently or coordinately with the OC2 protein. The OC2 3’ UTR can upregulate the transcription of PRC and can enforce translation by regulating PRC mRNAs via direct and indirect ceRNA sponging. OC2 has been shown to modulate PRC activity in lung adenocarcinoma cells (Ma Q et al., 2019). Additionally, PRC activity is increased in CRPC and is highly associated with Gleason grade (van Leenders et al., 2007; Berezovska et al., 2006). Thus, OC2 enforces novel, bimodal mechanisms of driving PRC previously undescribed before this work. The pathologic consequence of increased EZH2/PRC activity was next addressed using *in vitro* tumorigenic assays.

### 3.6 The ONECUT2 3’ UTR ceRNA network drives an aggressive phenotype

Gene set enrichment analysis indicated both OC2 protein and 3′ UTR networks drive EMT and metastasis. OC2 protein was previously shown to drive metastasis (Rotinen et al., 2018; Guo et al., 2019), but a role for the 3′UTR has not been addressed. We tested if the expression of the OC2 3′UTR alone could also increase metastatic potential. Because the enforcement of OC2 3′ UTR was transient and we relied on FAC sorting to maintain the expression, it was not adaptable to mouse models.

**FIGURE 6:**
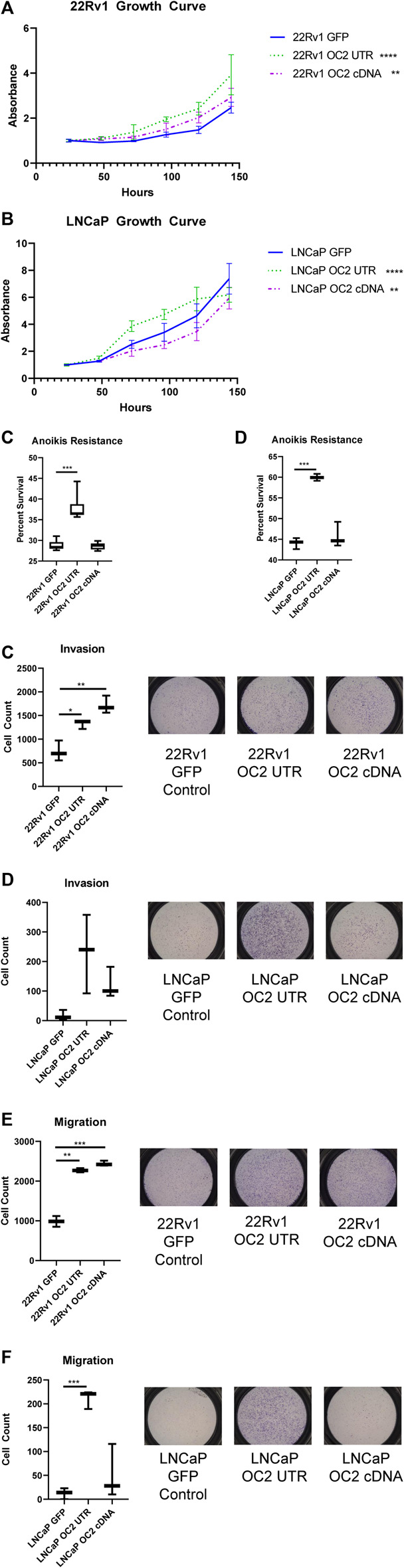
ONECUT2 3′ UTR network drives an aggressive phenotype. **(A)** Graph of 120-h growth curve 22Rv1 sublines. **(B)** Graph of 120-h growth curve LNCaP sublines. **(C)** Graph of 72-h anoikis assays in 22Rv1 sublines. **(D)** Graph of 72-h anoikis assays in LNCaP sublines. **(E)** (Right) Representative pictographs of invasion assays and (left) graph of counts 22Rv1 sublines. **(F)** (Right) Representative pictographs of invasion assays and (left) graph of counts LNCaP sublines. **(G)** (Right) Representative pictographs of migration assays and (left) graph of counts 22Rv1 sublines. **(H)** (Right) Representative pictographs of migration assays and (left) graph of counts LNCaP sublines (significance is determined using Welch’s T-test *p*-values *<.05, **<.005, ***<.0005, ****<.00005).

*In vitro* growth assays showed the OC2 3′ UTR sublines demonstrated increased proliferation than GFP control cells in both 22Rv1 and LNCaP backgrounds (Figures 6A, B). However, OC2 protein only promoted proliferation in 22Rv1 cells, while significantly lowering proliferation in LNCaP cells. Resistance to anoikis was tested using FAC-sorted cells; the OC2 protein induced no resistance to anoikis in either cell line (Figures 6C, D). However, OC2 3′UTR expression induced a significant increase in anoikis resistance in both cell backgrounds.

The potential of the OC2 3′ UTR to alter tumor invasion by degrading ECM was evaluated using a transwell invasion assay. In 22Rv1 cells, both OC2 protein and OC2 3′ UTR evoked greatly increased invasion over the GFP control (Figure 6E). In the LNCaP background, the OC2 3′ UTR showed greatly increased invasion potential, much higher than the also significantly increased OC2 protein (Figure 6F). The OC2 3’ UTR is capable of producing cells with an increased ability to degrade complex ECM structures, even surpassing the ability of the OC2 protein in these experiments.

A migratory simulation assay showed results that were similar to the invasion data. The OC2 protein and OC2 3′ UTR significantly increased the number of cells that migrated across the barrier (Figure 6G). However, in LNCaP cells, only OC2 3′ UTR was capable of increasing cell migration. Most LNCaP cells did not migrate during the course of the experiment. The invasion and migration results were surprising, in that the OC2 3′ UTR was more capable of promoting a more aggressive phenotype than the OC2 protein, and that LNCaP cells, a relatively indolent cell line, became much more aggressive when overexpressing the OC2 3′ UTR. Östling et al. (2011) examined direct AR modulation by miRNAs in prostate cancer cell lines and determined that AR had five functional mir-9 binding sites in the 3’ UTR and one in the coding sequence that were capable of lowering AR mRNA and protein. This data showed that the AR mRNA in 22Rv1 was more resistant to targeting than in LNCaP. Interestingly, miRDB target prediction between AR and the shortened variant ARV-7 indicate a loss of several mir-9 binding sites in the shorter form. This loss of mir-9 MREs may explain some of the variability in response between the ARV-7 harboring 22Rv1 and LNCaP which only has full length AR.

**FIGURE 7:**
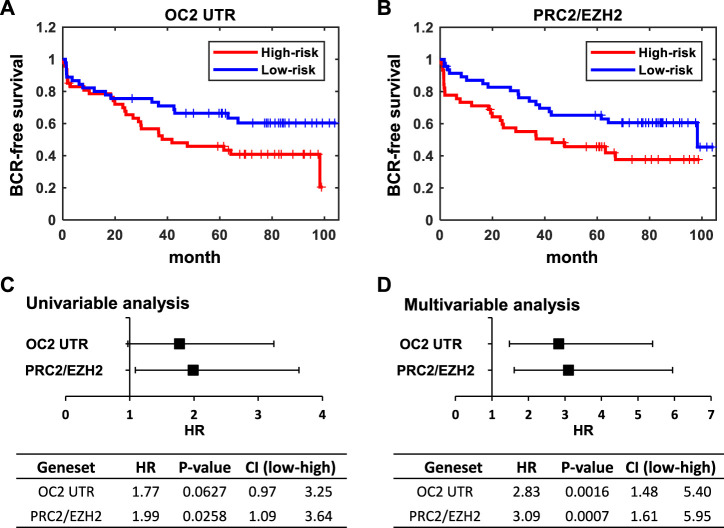
ONECUT2 3′ UTR network predicts poor outcomes. **(A)** Kaplan–Meier plot of BCR-free survival stratified by z-score of the OC2 3′ UTR signature. **(B)** Kaplan–Meier plot of BCR-free survival stratified by the z-score of the PRC2/EZH2 down signatures. The median of the z-score of each signature were used as cutoffs for the high-risk group and low-risk group. **(C)** Forest plot and table of hazard ratios estimated by using univariable analysis of the OC2 3′ UTR signature and PRC2/EZH2 down signature. **(D)** Forest plot and table of hazard ratios estimated by a multivariable analysis of the OC2 3′ UTR signature and PRC2/EZH2 down signature.

These results clearly indicate that the OC2 3′ UTR promotes metastatic features, including increased proliferation, invasion, migration, and anoikis resistance. The OC2 upregulated genes indicated networks that drove EMT, metastasis, and activation of EZH2. We then assessed if the aggressive OC2–PRC2 axis was also seen in patient populations. Using expression of OC2 ceRNA network genes in the Stockholm cohort data (GSE70769) and its clinical information, we performed survival analysis (Figure 7A). Patients were stratified into high-risk and low-risk groups at the median of z-score of OC2 UTR network genes. The high-risk group and low-risk groups show separation in the biochemical recurrence (BCR)-free survival rate. Additionally, we also checked the clinical association of the PRC2/EZH2 signature from the MsigDB (Liberzon et al., 2011). The PRC2/EZH2 signature also shows clear separation in the BCR-free survival rate (Figure 7B). The association with clinical outcome of the OC2 ceRNA network and PRC2/EZH2 signature was assessed by performing univariable and multivariable analysis (Figures 7C, D). Although the OC2 ceRNA network exhibits marginal significance in the univariable analysis, higher scores of OC2 ceRNA network and PRC2/EZH2 signature associate with poor clinical outcomes. The luciferase assays confirmed this PRC/EZH2 activation. EZH2 is associated with metastatic recurrence and lineage plasticity in prostate cancer patients (Wu et al., 2019; Cyrta et al., 202; Beltran et al., 2016). Taken together, OC2 3’ UTR mRNA is capable of promoting an aggressive phenotype that supports metastatic progression.

### 3.7 ONECUT2 3’ UTR as a modulator of the androgen axis

**FIGURE 8:**
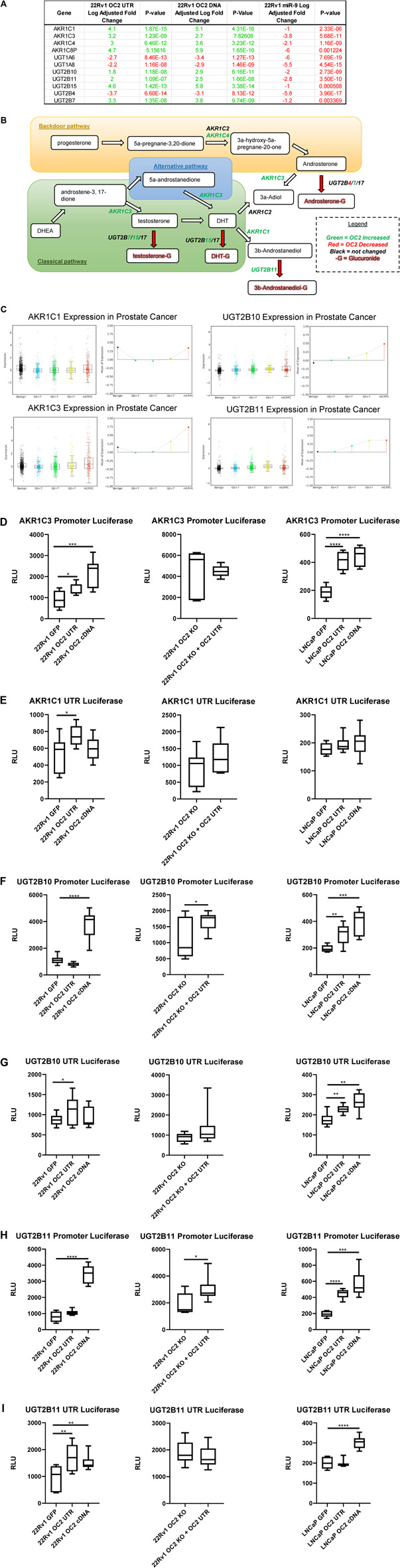
ONECUT2 3′ UTR modulates the androgen axis. **(A)** Microarray gene expression of the Aldo-Keto reductase family members and UDP-glucuronyl transferase genes. **(B)** Androgen synthesis pathway with relevant AKR/UGT enzymes, their directionality, their activity, and their OC2-driven expression. **(C)** (Top left) AKR1C1 gene expression from the Prostate Cancer Transcriptome Atlas (PCTA) sorted by cancer grade benign to mCRPC, (bottom left) AKR1C3 gene expression from the Prostate Cancer Transcriptome Atlas (PCTA) sorted by cancer grade benign to mCRPC, (top right) UGT2B10 gene expression from the Prostate Cancer Transcriptome Atlas (PCTA) sorted by cancer grade benign to mCRPC, and (bottom right) UGT2B11 gene expression from the Prostate Cancer Transcriptome Atlas (PCTA) sorted by cancer grade benign to mCRPC. OC2 induces significant AKR1C3 transcriptional **(D)** AKR1C3 promoter luciferase activity graphs, 22Rv1 sublines (left), 22Rv1 OC2 knockout (center), and LNCaP (right). **(E)** AKR1C1 3′ UTR luciferase activity graphs, 22Rv1 (left), 22Rv1 OC2 knockout (center), and LNCaP sublines (right) (significance determined using Welch’s T-test *p*-values *<.05, **<.005, ***<.0005, ****<.00005).

The OC2 network upregulated a number of enzymes related to the androgen pathway (Figures 8A, B). The Aldo-Keto reductase family members (*AKR1C1*, *AKR1C3*, *AKR1C4*, and *AKR1C8P*) *AKR1C1* and *AKR1C3* were among the highest upregulated genes in our OC2 network, and their expression increases with disease progression in prostate tumors (Figure 8C, left). In addition, *AKR1C1* and *AKR1C3* were among the lowest downregulated genes by the overexpression of miR-9. AKR1C1 and AKR1C3 have both been shown to be greatly increased in bone marrow metastases when compared to prostate cancer primary tumor sites (Stanbrough et al., 2006). AKR1C3 has also been shown to be upregulated in response to androgen deprivation therapy in CRPC (Adeniji et al., 2013)*.* AKR1C1 and AKR1C2 reduce the potent androgen DHT to 3β-androstanediol and 3α-androstanediol, respectively (Steckelbroeck et al., 2004)*.* AKR1C3 can also function as a coactivator of AR by binding to dimerized and phosphorylated androgen receptors (Zeng et al., 2017). Lastly, AKR1C3 was shown to drive resistance to the antiandrogen therapy drug abiraterone (Liu et al., 2017). The UDP-glucuronyl transferase genes *UGT2B7*, *UGT2B10*, and *UGT2B11* were upregulated by the OC2 network, and in particular, *UGT2B10* and *UGT2B11* were among the most downregulated genes generated by the overexpression of miR-9. The expression of *UGT2B10* and *UGT2B11* correlates with disease progression in prostate cancer tumors (see Figure 8C, right). UGT2B7 was previously found in glucuronidate DHT, testosterone, and androsterone, thus irreversibly inactivating these androgens (Chouinard et al., 2008). UGT2B11 was found to conjugate 3b-androstanediol and weakly conjugate other androgens (Jin et al., 1997). Currently it is not known if the UGT2B10 enzyme can conjugate androgen substrates. Luciferase activity experiments were performed in the same dual-luciferase system as the PRC complex members. OC2 protein and 3′ UTR were able to increase AKR1C3 promoter activity in both 22Rv1 and LNCaP cell lines. This increase of activity was dependent on the OC2 protein because the KO of the OC2 protein abrogated the increased promoter activity even when the 3′ UTR was overexpressed (Figure 8D, left, center, and right). The AKR1C1 3′ UTR showed increased activity, but only in the 22Rv1 cell background and only a modest but significant increase (Figure 8E, left, center, and right). OC2 was able to modulate the promoter activity of UGT2B10 in LNCaP, but only the OC2 protein was capable of significantly increasing UGT2B10 activity in the 22Rv1 cell background. The overexpression of the OC2 3′ UTR in the absence of the OC2 protein yielded a minor increase in promoter activity (Figure 8F). The overexpression of the OC2 3′ UTR produced more translational activity of UGT2B10 in both 22Rv1 and LNCaP backgrounds but the protein was only able to significantly increase activity in LNCaP cells (Figure 8G). The UGT2B11 promoter activity response to OC2 overexpression was similar to the UGT2B10 promoter; OC2 3′ UTR was unable to increase activity in the 22Rv1 cells, but both the protein and UTR were capable of significantly increasing promoter activity in LNCaP. In the OC2 knockout cells, there was minimal increase in promoter activity in response to 3′ UTR overexpression (Figure 8H). The modulation of the UGT2B11 3′ UTR luciferase activity was seen by both protein and 3′ UTR in 22Rv1. However, activity in LNCaP was only increased by the OC2 protein. There was no difference in the OC2 protein knockout indicating that the UGT2B11 activity increase is dependent on OC2 protein (Figure 8I). We assayed a number of lncRNAs known to increase AR activity to see if the OC2 protein or 3’ UTR was altering the level of these lncRNAs. While many lncRNAs saw statistically significant downregulation the magnitudes of the changes were modest (Supplementary Figure S8A). It has been previously shown that OC2 drives mechanisms of androgen independence (Rotinen et al., 2018; Guo et al., 2019). Our data here show new methods by which OC2 can alter androgen synthesis by increasing Aldo-Keto reductase and UDP-glucuronyl transferase genes within prostate cancer cells.

## 4 Discussion

This study has demonstrated that the unusually long, highly evolutionarily conserved 3′ UTR of OC2 has an autonomous function, distinct from but partially overlapping with the OC2 protein. 3′ UTR of OC2 is both the fifth longest and has the fifth most miRNA-binding sites of any mRNA. OC2 3’ UTR structurally is a highly complex macromolecule that makes it well suited to be a miRNA sponge capable of sequestering many miRNAs.

We performed multiple computational analyses that indicated that tumors overexpressing OC2 should drive a network that is enriched for tumor growth and neuronal development genes. This network includes a subset of enriched miRNA effectors and these mRNAs were expressed at higher levels in prostate cancer tumors than normal prostate tissue. Genes that were positively correlated that shared MREs with OC2 were significantly enriched over genes without shared MREs.

The OC2 3′ UTR has many miR-9 sites, but paradoxically the mRNA is resilient to targeting by miR-9, as assessed by multiple assays, in addition to the finding of coordinate expression within global human tissues. This resilience indicated that OC2 mRNA is insensitive to some miRNA that bind to the OC2 mRNA. Our study confirms that OC2 3’ UTR acts as a master ceRNA as suggested by (Sarver & Subramanian, 2012).

The overexpression of the OC2 protein and OC2 3′ UTR drove overwhelmingly similar gene networks that were analogous to our *in silico* ceRNA model. Additionally, gene set enrichment analysis of OC2 3′ UTR gene expression indicated that the 3′ UTR ceRNA network was enriched for increased EZH2/PRC activity and metastatic promoting genes. Luciferase data indicate that the OC2 protein and mRNA act coordinately to increase PRC activity. In addition, the data showed that OC2 uses multiple mechanisms to increase EZH2 activity. However, it should be emphasized that EZH2 has PRC-independent activity in prostate cancer where EZH2 is phosphorylated to act as a transcriptional coactivator (Xu et al., 2012). The increase of EZH2 luciferase activity we showed could not distinguish between these two capabilities. Our results clearly show that the OC2 3′ UTR network drives a metastasis-promoting phenotype, in some cases with even a greater effect than the OC2 protein. The 3’ UTR supports EMT and drives strong migration, invasion, and resistance to anoikis effects.

The effects of the pro-metastatic OC2 network were seen in patient data, as the OC2 3′UTR network genes were significantly predictive of biochemical recurrence in prostate cancer. The OC2 protein and 3′ UTR both increased gene expression of androgen modifying enzymes in opposition to our miR-9 overexpression cells, which is consistent with the previous published findings that miR-9 increases AR activity in LNCaP cells (Fletcher et al., 2019)*.* The OC2-driven mRNA increases of Aldo-Keto reductase and UDP-glucuronyl transferase were confirmed using luciferase activity assays. These enzymes are implicated in the modification of the classical, backdoor, and alternate androgen synthesis pathways (Mostaghel et al., 2016; Fiandalo et al., 2018; Jin & Penning, 2001). The OC2 effects on androgen synthesis need to be further validated using androgen metabolism analysis methods. Our data appear to show, through upregulation of enzymes, androgens are shunted towards 3β-androstanediol and then glucoronidated to permanently remove them from the cell. The enzymatic directionality of the OC2-induced enzymes (Figure 8B) shows a metabolic preference against 3β-androstanediol which is antiproliferative in prostate cancer (Weihua et al., 2002) and towards 3α-androstanediol which interestingly acts as neurosteriod (Reddy, 2004).

The finding that a protein and its 3′ UTR drive similar gene networks to increase tumor aggressiveness has not been previously described in the literature and represents a novel self-reinforcing transcriptional mechanism in both genetics and in cancer. This evolutionary conserved signal redundancy may present a new limitation to cancer therapy as molecules to inhibit OC2 protein maybe unable to completely nullify these coordinated effects. These data also show weakness in 3′ UTR-testing systems that rely on using short 3′ UTR fragments to model the effect of miRNA gene regulation on very long 3’ UTR containing transcripts. The distinct role of the OC2 transcription factor protein in developmental differentiation is evolutionarily conserved. Likewise, the OC2 3′UTR is conserved, potentially to reinforce and preserve the same differentiation signals. Our data suggest that the OC2 mRNA is a powerful ceRNA capable of producing an aggressive tumor phenotype. Activation of OC2 in prostate cancer likely harnesses this powerful bimodal mechanism to drive lethal disease.

## Data Availability

The original contributions presented in the study are publicly available. This data can be found here: NCBI repository - Accession Number GSE235003.
