# Supplementary material for: Autonomous action and cooperativity between the ONECUT2 transcription factor and its 3′ untranslated region
Source: Front Cell Dev Biol. 2023 Jul 5;11:1206259. doi: 10.3389/fcell.2023.1206259 (PMC10356556; doi:10.3389/fcell.2023.1206259)
Supplement: Supplementary file 1 [file Presentation1.pdf]

Supplemental Figure 1

A Mammalian Conservation of the ONECUT2 3'UTR sequence

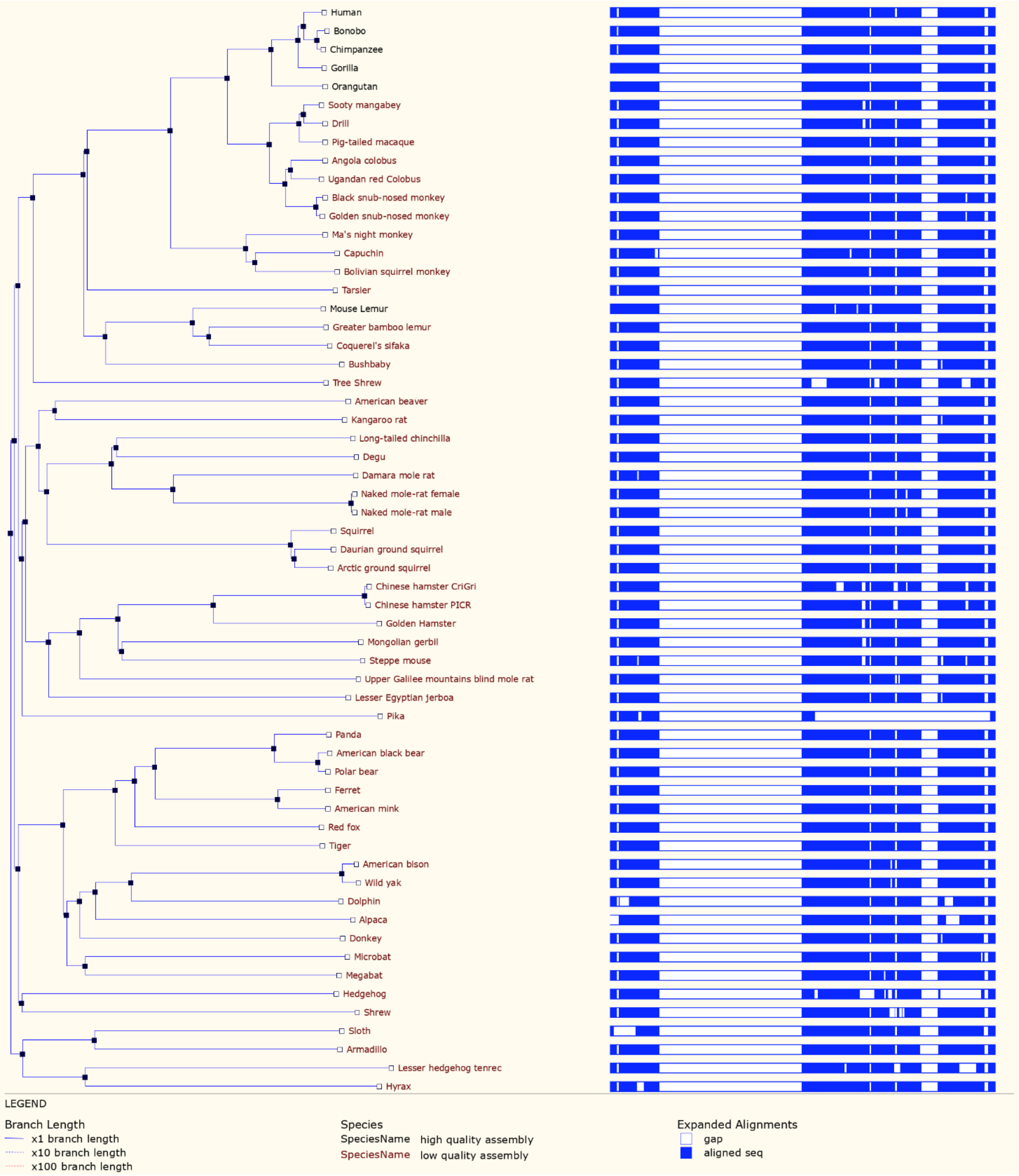

(A) Sequence alignments of the proximal portion ONECUT2 3' UTR mRNA in selected mammals.

Supplemental Figure 1

B Mammalian Conservation of the ONECUT2 3'UTR sequence

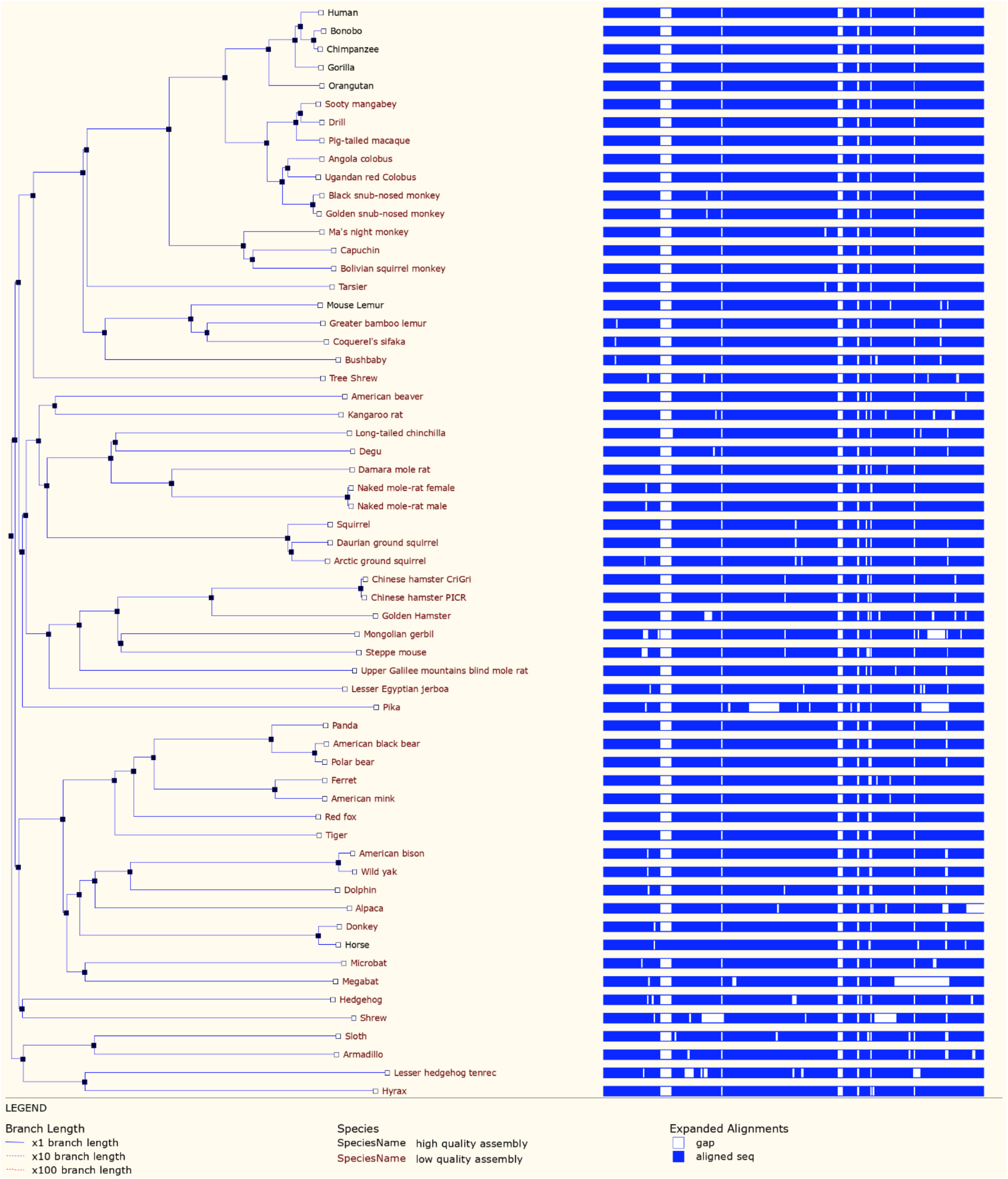

(B) Sequence alignments of the distal portion ONECUT2 3' UTR mRNA in selected mammals.

Supplemental Figure 2

A

No	Dataset name	Journal	Number of samples	Number of samples per Grade	Reference
1	SU2C/PCF Dream Team	PNAS 2019	270	GS<7 (18), GS=7 (64), GS>7 (134)	PMID: 31061129
2	Neuroendocrine PC	Nat Med 2016	114	mCRPC (49), paired normal (49)	PMID: 26855148
3	Broad/Cornell	Nat Genet 2012	31	GS<7 (4), GS=7 (14), GS>7 (2)	PMID: 22610119
4	Fred Hutchinson CRC	Nat Med 2016	171	22 Primary, 149 Metastasis	PMID: 26928463
5	MSKCC	Cancer Cell 2010	156	GS<7 (41), GS=7 (76), GS>7 (22)	PMID: 20579941
6	TCGA PRAD Firehouse		498	GS<7 (45), GS=7 (247), GS>7 (206)	https://www.cancer.gov/tcga

B

Association of combined correlation score & Spearman’s Rho

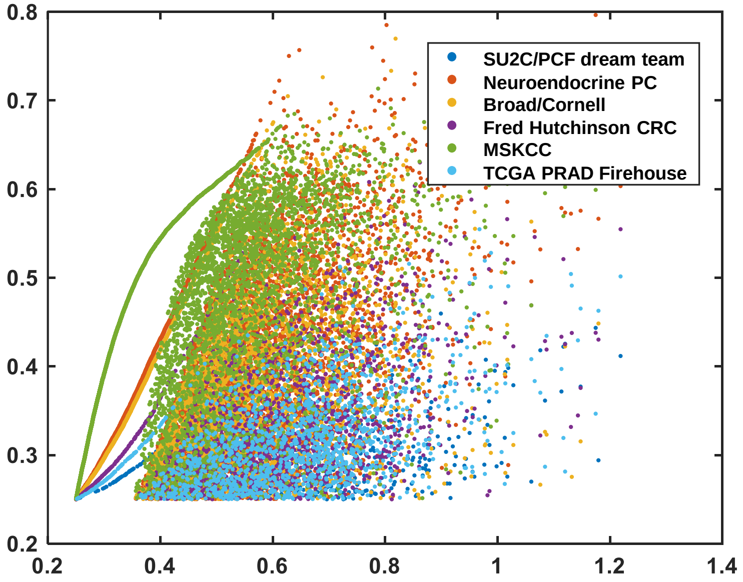

(A) Table describing the datasets used to produce the OC2 ceRNA network. (B) The association of combined score and Spearman’s Rho of OC2 and OC2 high-correlated genes in each dataset. The color of dots represents the datasets.

Supplemental Figure 2

C

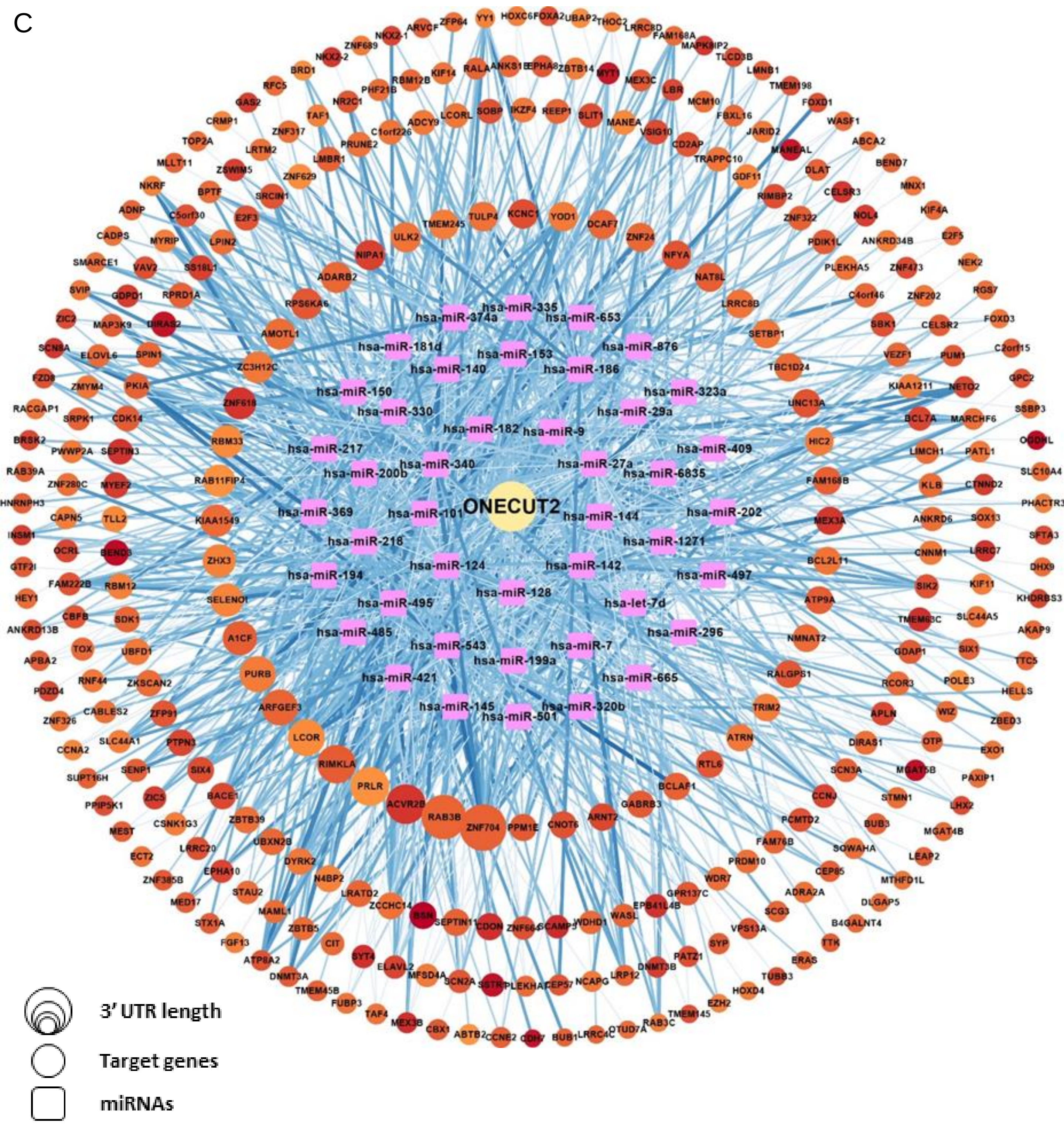

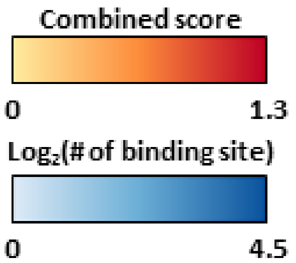

(C) The ONECUT2 ceRNA network. The circular nodes are OC2 high correlated genes that have miRNA binding sites. The rectangle nodes are miRNAs. Node size represents the length of 3' UTR. Node color represents the combined score. Edges show the miRNA-target binding information. The color and width of edge represent the number of binding sites.

Supplemental Figure 2

D

ONECUT2 3’ UTR conserved miR-9 binding sites

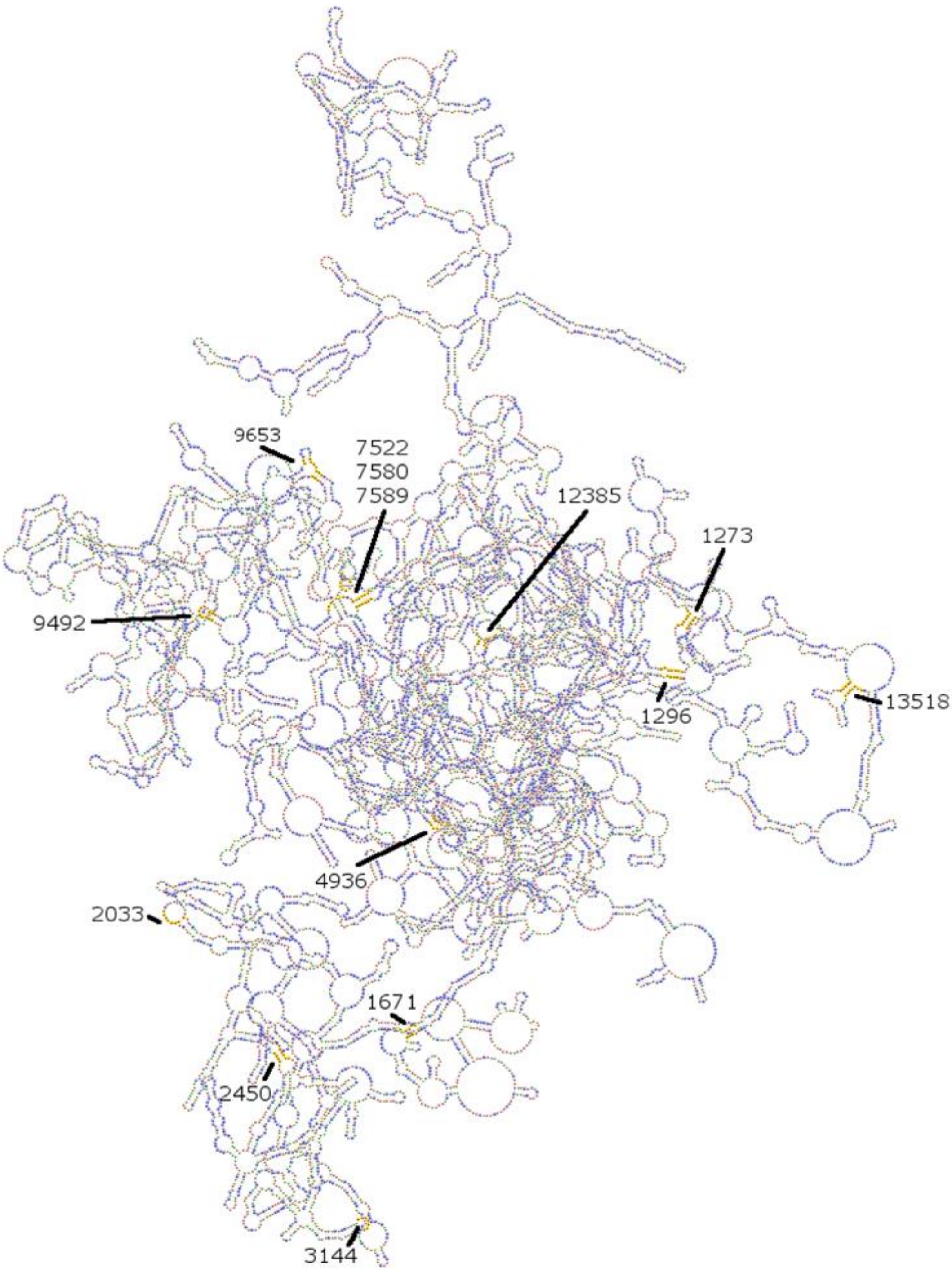

(D) Schematic showing the highly complex structure of the 3’ UTR denoting the locations of the most conserved miR-9 binding sites or microRNA Response Elements (MREs). Indicating that many MREs are not readily accessible and would be difficult to dock into the RISC (The RNA-Induced Silencing Complexes) protein assemblies.

Supplemental Figure 2

F

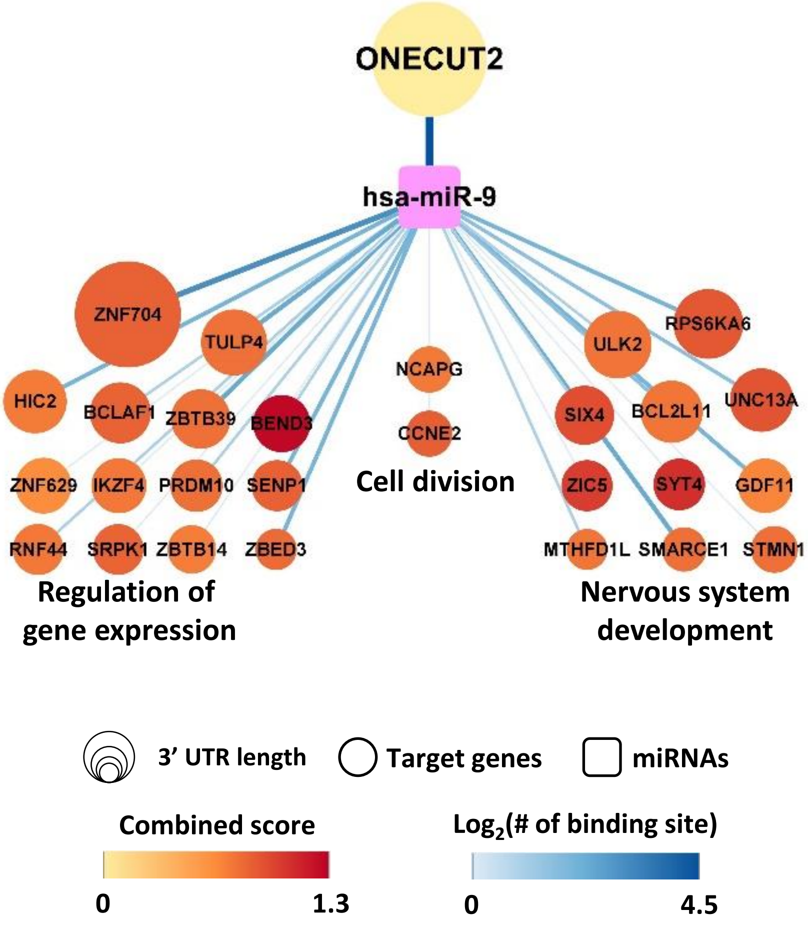

(F) The subnetwork of OC2-miR-9-targets.

Figure 4 Supplemental

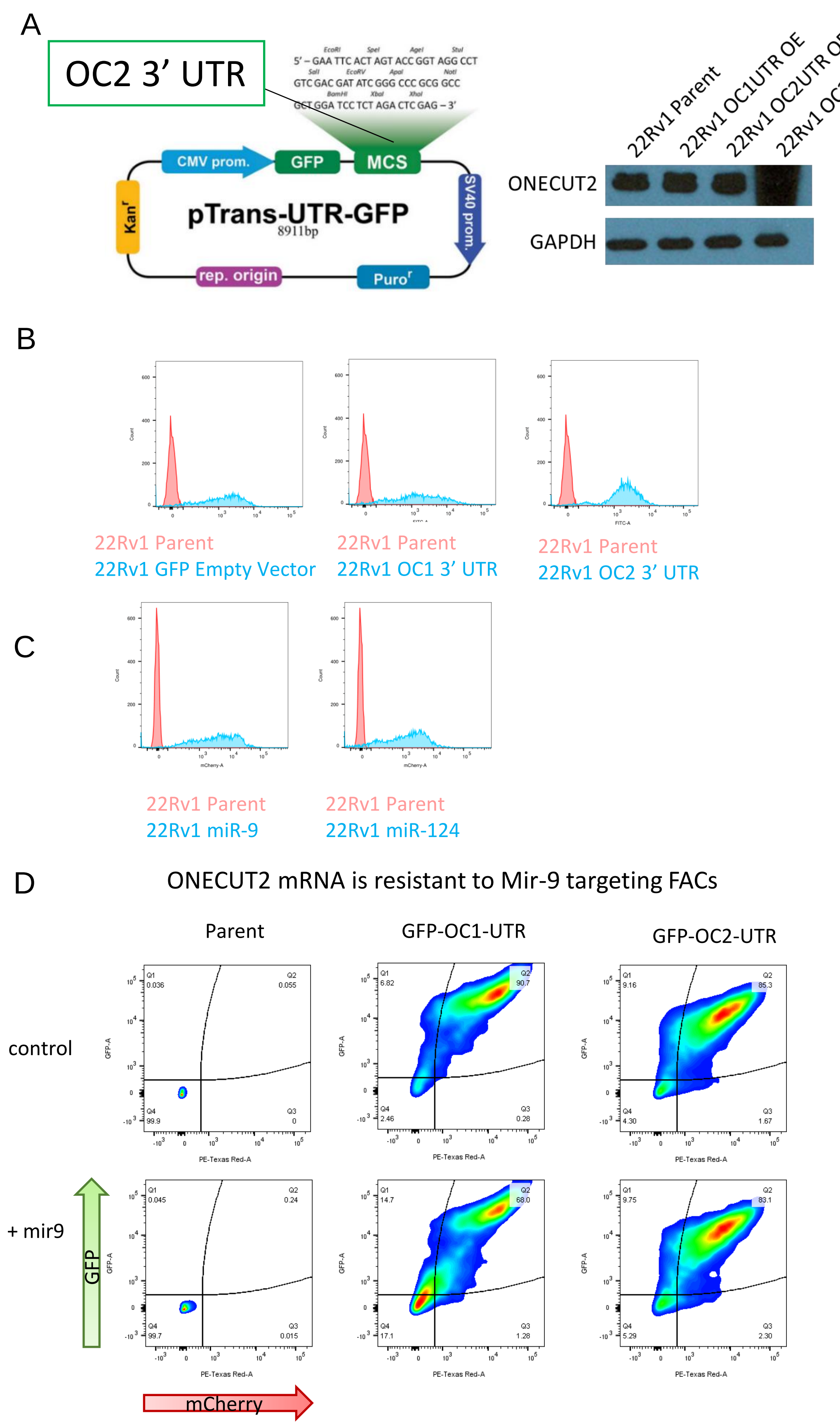

(A, Left) Vector used to select for OC2 3' UTR overexpressing cells. Overexpression of OC1 3' UTR and OC2 3' UTR did not increase OC2 protein expression.
(A, Right) Capture of autoradiograph from Western blot showing ONECUT2 protein in 22Rv1 parent, after overexpression of the OC1 3' UTR, overexpression of OC2 3' UTR and OC2 cDNA overexpression.
(B) Flow Cytometric histograms of FITC (Fluorescein isothiocyanate) positivity compared to parental fluorescence from GFP reporters in GFP Empty Vector controls cells (Left), OC1 3' UTR overexpressing (Center) and OC1 3' UTR overexpressing (Right).
(C) Flow Cytometric histograms of mCherry positivity compared to parental fluorescence from mCherry reporters in miR-9 overexpressing (Left) and miR-124 overexpressing (Right).
(D) 22Rv1 Parent, Dual labeled Empty Cherry/GFP- OC1 UTR and Empty Cherry/GFP-OC2 UTR cells were subjected to miR-9 treatment for 24 hours (Bottom row) loss of GFP positive cells (Top right quadrants) was greater in OC1 UTR than in OC2 UTR stronger miR-9 repression in OC1 and minimal repression of OC2 UTR.

Figure 4 Supplemental

E

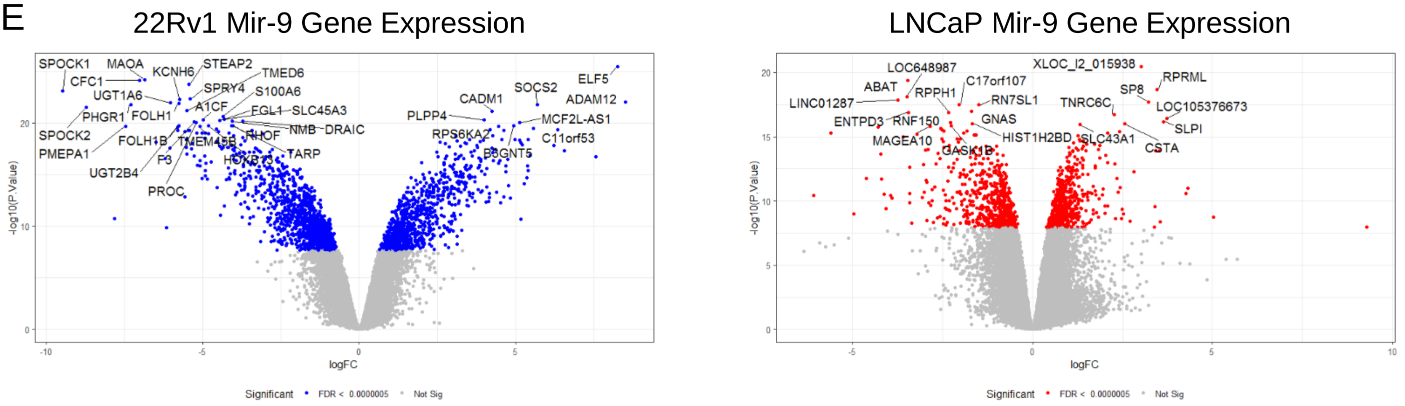

(E) Volcano plots of significantly differentially expressed genes in miR-9 overexpressing cells 22Rv1 Left, LNCaP Right. (Significance = FDR < 0.0000005).

Figure 4 Supplemental

F

CELL LINE	GO BIO 2018 Term	P-value
22Rv1 OC2 cDNA	extracellular matrix organization (GO:0030198)	1.18E-06
22Rv1 OC2 cDNA	diterpenoid metabolic process (GO:0016101)	1.66E-06
22Rv1 OC2 cDNA	retinoid metabolic process (GO:0001523)	1.77E-06
22Rv1 OC2 cDNA	lipid transport (GO:0006869)	1.39E-05
22Rv1 OC2 cDNA	extracellular matrix disassembly (GO:0022617)	1.66E-05
22Rv1 OC2 cDNA	bile acid and bile salt transport (GO:0015721)	2.05E-05
22Rv1 OC2 cDNA	monocarboxylic acid transport (GO:0015718)	2.13E-05
22Rv1 OC2 cDNA	negative regulation of cellular process (GO:0048523)	5.16E-05
22Rv1 OC2 cDNA	organic hydroxy compound transport (GO:0015850)	7.20E-05
22Rv1 OC2 cDNA	synapse assembly (GO:0007416)	8.76E-05
22Rv1 OC2 UTR	adherens junction organization (GO:0034332)	4.64E-05
22Rv1 OC2 UTR	generation of neurons (GO:0048699)	1.49E-04
22Rv1 OC2 UTR	negative regulation of protein kinase activity (GO:0006469)	1.52E-04
22Rv1 OC2 UTR	Rho protein signal transduction (GO:0007266)	1.85E-04
22Rv1 OC2 UTR	extracellular matrix organization (GO:0030198)	1.87E-04
22Rv1 OC2 UTR	neuron migration (GO:0001764)	2.29E-04
22Rv1 OC2 UTR	negative regulation of STAT cascade (GO:1904893)	2.63E-04
22Rv1 OC2 UTR	calcium-dependent cell-cell adhesion via plasma membrane cell adhesion molecules (GO:0016339)	2.63E-04
22Rv1 OC2 UTR	negative regulation of coagulation (GO:0050819)	2.65E-04
22Rv1 OC2 UTR	neuron projection morphogenesis (GO:0048812)	2.81E-04

G

CELL LINE	GO BIO 2018 Term	P-value
LNCaP OC2 cDNA	type I interferon signaling pathway (GO:0060337)	1.35E-06
LNCaP OC2 cDNA	cellular response to type I interferon (GO:0071357)	1.35E-06
LNCaP OC2 cDNA	positive regulation of dendrite development (GO:1900006)	1.54E-04
LNCaP OC2 cDNA	negative regulation of viral genome replication (GO:0045071)	8.74E-04
LNCaP OC2 cDNA	cytokine-mediated signaling pathway (GO:0019221)	0.001113
LNCaP OC2 cDNA	positive regulation of cell death (GO:0010942)	0.001432
LNCaP OC2 cDNA	negative regulation of viral life cycle (GO:1903901)	0.0018424
LNCaP OC2 cDNA	cGMP-mediated signaling (GO:0019934)	0.0019672
LNCaP OC2 cDNA	myelin maintenance (GO:0043217)	0.0019672
LNCaP OC2 cDNA	regulation of viral genome replication (GO:0045069)	0.0020754
LNCaP OC2 UTR	nervous system development (GO:0007399)	8.71E-11
LNCaP OC2 UTR	synapse assembly (GO:0007416)	1.91E-10
LNCaP OC2 UTR	cell morphogenesis involved in neuron differentiation (GO:0048667)	3.34E-06
LNCaP OC2 UTR	calcium-dependent cell-cell adhesion via plasma membrane cell adhesion molecules (GO:0016339)	7.13E-06
LNCaP OC2 UTR	axonogenesis (GO:0007409)	7.58E-06
LNCaP OC2 UTR	chemical synaptic transmission (GO:0007268)	1.12E-05
LNCaP OC2 UTR	modulation of chemical synaptic transmission (GO:0050804)	1.65E-05
LNCaP OC2 UTR	branching involved in ureteric bud morphogenesis (GO:0001658)	1.91E-05
LNCaP OC2 UTR	ureteric bud morphogenesis (GO:0060675)	1.91E-05
LNCaP OC2 UTR	positive regulation of mesonephros development (GO:0061213)	1.92E-05

(F) Biological process gene ontology of significantly overexpressed genes in 22Rv1 cells by OC2 protein (Green/upper) and OC2 3’ UTR (Blue/lower). (G) Biological process gene ontology of significantly overexpressed genes in LNCaP cells by OC2 protein (Red/upper) and OC2 3’ UTR (Orange/lower).

Figure 4 Supplemental

H

CELL LINE	ARCH4 Transcription Factor Prediction	P-value
22Rv1 OC2 cDNA	HNF4A human tf ARCHS4 coexpression	3.11E-17
22Rv1 OC2 cDNA	FOXA3 human tf ARCHS4 coexpression	9.97E-16
22Rv1 OC2 cDNA	NR1H4 human tf ARCHS4 coexpression	2.83E-14
22Rv1 OC2 cDNA	KLF5 human tf ARCHS4 coexpression	1.57E-11
22Rv1 OC2 cDNA	HNF4G human tf ARCHS4 coexpression	1.57E-11
22Rv1 OC2 cDNA	ISX human tf ARCHS4 coexpression	1.57E-11
22Rv1 OC2 cDNA	MST1R human tf ARCHS4 coexpression	1.57E-11
22Rv1 OC2 cDNA	VDR human tf ARCHS4 coexpression	6.98E-11
22Rv1 OC2 cDNA	GATA5 human tf ARCHS4 coexpression	6.98E-11
22Rv1 OC2 cDNA	CREB3L3 human tf ARCHS4 coexpression	6.98E-11
22Rv1 OC2 cDNA	**ONECUT2 human tf ARCHS4 coexpression**	7.93E-05
22Rv1 OC2 UTR	HNF4A human tf ARCHS4 coexpression	1.42E-10
22Rv1 OC2 UTR	NPAS3 human tf ARCHS4 coexpression	3.19E-09
22Rv1 OC2 UTR	ZMAT4 human tf ARCHS4 coexpression	3.19E-09
22Rv1 OC2 UTR	EBF3 human tf ARCHS4 coexpression	1.42E-08
22Rv1 OC2 UTR	NR1I2 human tf ARCHS4 coexpression	1.42E-08
22Rv1 OC2 UTR	FOXA3 human tf ARCHS4 coexpression	1.42E-08
22Rv1 OC2 UTR	MEOX2 human tf ARCHS4 coexpression	6.03E-08
22Rv1 OC2 UTR	VDR human tf ARCHS4 coexpression	6.03E-08
22Rv1 OC2 UTR	LHX9 human tf ARCHS4 coexpression	2.45E-07
22Rv1 OC2 UTR	PBX3 human tf ARCHS4 coexpression	2.45E-07
22Rv1 OC2 UTR	**ONECUT2 human tf ARCHS4 coexpression**	2.83E-03

I

CELL LINE	ARCH4 Transcription Factor Prediction	P-value
LNCaP OC2 cDNA	HOXD1 human tf ARCHS4 coexpression	1.11E-03
LNCaP OC2 cDNA	NR2E3 human tf ARCHS4 coexpression	4.28E-03
LNCaP OC2 cDNA	MEOX2 human tf ARCHS4 coexpression	4.28E-03
LNCaP OC2 cDNA	ONECUT2 human tf ARCHS4 coexpression	4.28E-03
LNCaP OC2 cDNA	INSM1 human tf ARCHS4 coexpression	0.0042827
LNCaP OC2 cDNA	GBX2 human tf ARCHS4 coexpression	0.0042827
LNCaP OC2 cDNA	NHLH2 human tf ARCHS4 coexpression	0.0042827
LNCaP OC2 cDNA	NFATC4 human tf ARCHS4 coexpression	0.0146221
LNCaP OC2 cDNA	ELK3 human tf ARCHS4 coexpression	0.0146221
LNCaP OC2 cDNA	SNAI1 human tf ARCHS4 coexpression	0.0146221
LNCaP OC2 UTR	MYT1 human tf ARCHS4 coexpression	2.83E-15
LNCaP OC2 UTR	DPF1 human tf ARCHS4 coexpression	1.71E-12
LNCaP OC2 UTR	NPAS3 human tf ARCHS4 coexpression	1.71E-12
LNCaP OC2 UTR	SCRT1 human tf ARCHS4 coexpression	1.89E-11
LNCaP OC2 UTR	ZSCAN18 human tf ARCHS4 coexpression	1.89E-11
LNCaP OC2 UTR	ETV1 human tf ARCHS4 coexpression	6.07E-11
LNCaP OC2 UTR	BARHL2 human tf ARCHS4 coexpression	6.07E-11
LNCaP OC2 UTR	MYT1L human tf ARCHS4 coexpression	6.07E-11
LNCaP OC2 UTR	TUB human tf ARCHS4 coexpression	6.07E-11
LNCaP OC2 UTR	POU6F1 human tf ARCHS4 coexpression	1.91E-10
LNCaP OC2 UTR	**ONECUT2 human tf ARCHS4 coexpression**	**2.89E-07**

(H). ONECUT protein and 3‘ UTR gene networks drive comparable transcriptional activities
Table K. All RNA-seq and ChIP-seq Sample and Signature Search (ARCHS4) analysis-implicated highly significant transcription factors in 22Rv1 cells by OC2 protein (Green/upper) and OC2 3' UTR (Blue/lower) (OC2 Transcription Factor in BOLD). (I) ARCHS4 analysis-implicated highly significant transcription factors in LNCaP cells by OC2 protein (Red/upper) and OC2 3' UTR (Orange/lower) (OC2 Transcription Factor in BOLD).

Figure 4 Supplemental

J

CELL LINE	Term	P-value
22Rv1 OC2 cDNA	SUZ12 CHEA	1.52E-33
22Rv1 OC2 cDNA	EZH2 CHEA	5.65E-07
22Rv1 OC2 cDNA	AR CHEA	1.97E-05
22Rv1 OC2 cDNA	SUZ12 ENCODE	0.00104126
22Rv1 OC2 cDNA	SOX2 CHEA	0.00146673
22Rv1 OC2 cDNA	ESR1 CHEA	0.00199668
22Rv1 OC2 cDNA	REST ENCODE	0.00224875
22Rv1 OC2 cDNA	NANOG CHEA	0.0029912
22Rv1 OC2 cDNA	TRIM28 CHEA	0.00375575
22Rv1 OC2 cDNA	EZH2 ENCODE	0.00546224
22Rv1 OC2 UTR	SUZ12 CHEA	2.83E-28
22Rv1 OC2 UTR	EZH2 CHEA	3.46E-07
22Rv1 OC2 UTR	SUZ12 ENCODE	2.09E-05
22Rv1 OC2 UTR	AR CHEA	1.93E-04
22Rv1 OC2 UTR	SALL4 CHEA	9.97E-04
22Rv1 OC2 UTR	ESR1 CHEA	0.00228353
22Rv1 OC2 UTR	EZH2 ENCODE	0.00367934
22Rv1 OC2 UTR	REST CHEA	0.00413162
22Rv1 OC2 UTR	SOX2 CHEA	0.00421559

CELL LINE	Term	P-value
LNCaP OC2 cDNA	SUZ12 CHEA	5.96E-10
LNCaP OC2 cDNA	TP53 CHEA	0.00627167
LNCaP OC2 cDNA	AR CHEA	0.02565141
LNCaP OC2 cDNA	ESR1 CHEA	0.04338277
LNCaP OC2 UTR	SUZ12 CHEA	2.19E-43
LNCaP OC2 UTR	REST ENCODE	7.66E-25
LNCaP OC2 UTR	EZH2 CHEA	1.60E-06
LNCaP OC2 UTR	SALL4 CHEA	2.51E-06
LNCaP OC2 UTR	REST CHEA	3.64E-06
LNCaP OC2 UTR	SUZ12 ENCODE	1.18E-05
LNCaP OC2 UTR	TRIM28 CHEA	3.83E-05
LNCaP OC2 UTR	EZH2 ENCODE	1.72E-04
LNCaP OC2 UTR	SOX2 CHEA	3.27E-04
LNCaP OC2 UTR	NANOG CHEA	0.00510886

K

MicroRNAs implicated by target upregulation in the overlap genes between OC-UTR and OC2-CDNA (22Rv1)

Index	miRNA	Z-score	Combined Score
1	hsa-miR-6867-5p	-3.939183611	15.00888021
2	hsa-miR-4444	-2.68657346	14.62580958
3	**hsa-miR-124-3p**	-8.296233762	14.43249656
4	hsa-miR-190a-3p	-4.728759082	13.25230593
5	hsa-miR-494-5p	-3.219074876	12.98190532
6	hsa-miR-376b-3p	-2.320152469	11.64835737
7	hsa-miR-574-5p	-3.501389274	11.3448227
8	hsa-miR-3646	-2.715478516	10.755879
9	hsa-miR-5011-5p	-4.536434673	10.29440577
10	hsa-miR-4719	-2.301232673	10.24113387
11	hsa-miR-3662	-2.774412235	9.663078637
12	**hsa-miR-9-5p**	-3.003646107	9.039921592
13	hsa-miR-4698	-2.541281264	8.960740758
14	hsa-miR-1238-3p	-2.137010752	8.601269422
15	hsa-miR-6817-3p	-3.005172401	8.58974375

(J) ChIP-X Enrichment Analysis (ChEA) and ENCODE transcription factor ChIP-seq databases analysis to determine downstream transcription factors: 22Rv1 OC2 protein (Green), 22Rv1 OC2 3' UTR (Blue), LNCaP OC2 protein (Red) and LNCaP OC2 3' UTR (Orange). (K) miRNAs predicted to target OC2 upregulated network genes.

Figure 4 Supplemental

L

ONECUT2 cDNA/UTR Gene Expression

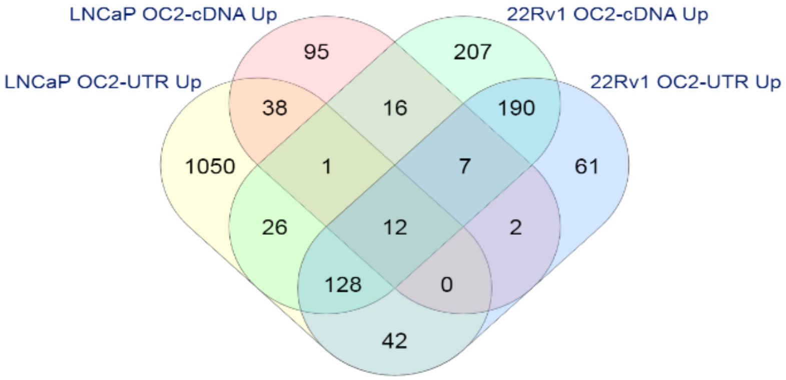

M

1 22Rv1 ONECUT2 OC2-cDNA

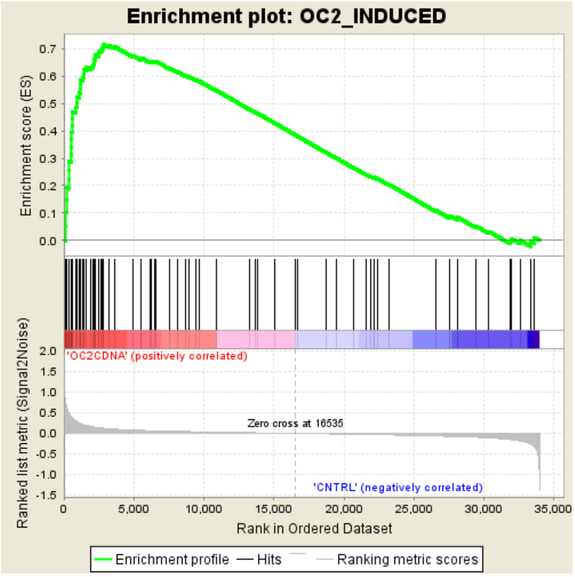

2 22Rv1 ONECUT2 OC2-UTR

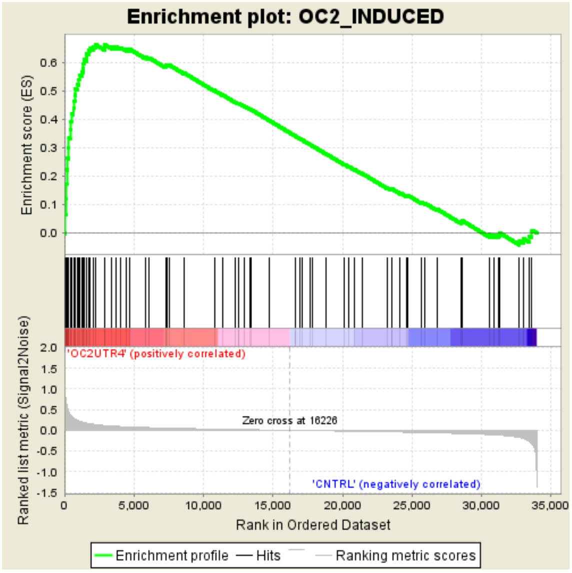

N

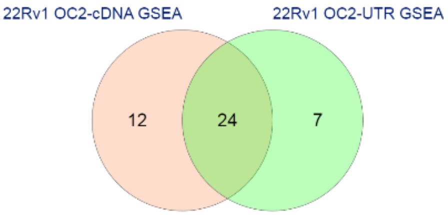

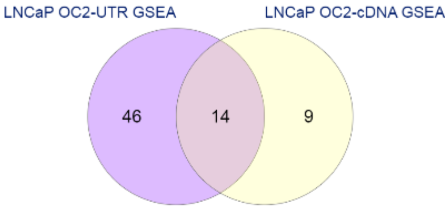

(L) A four-way Venn diagram of all LNCaP OC2cDNA/3'UTR and 22Rv1 OC2cDNA/3'UTR significantly upregulated genes. (M) (1 and 2) Gene Set Enrichment Analysis of both OC2 protein and 3' UTR networks have significant concordance with previous published OC2 protein regulated genes in prostate cancer models

(N, Left). The overlap of significantly enriched Hallmarks & KEGG canonical pathways for 22Rv1 OC2 protein/OC2 3'UTR. (N, Right) The overlap of significantly enriched Hallmarks & KEGG canonical pathways for LNCaP OC2 protein/OC2 3'UTR.

Figure 4 Supplemental

O

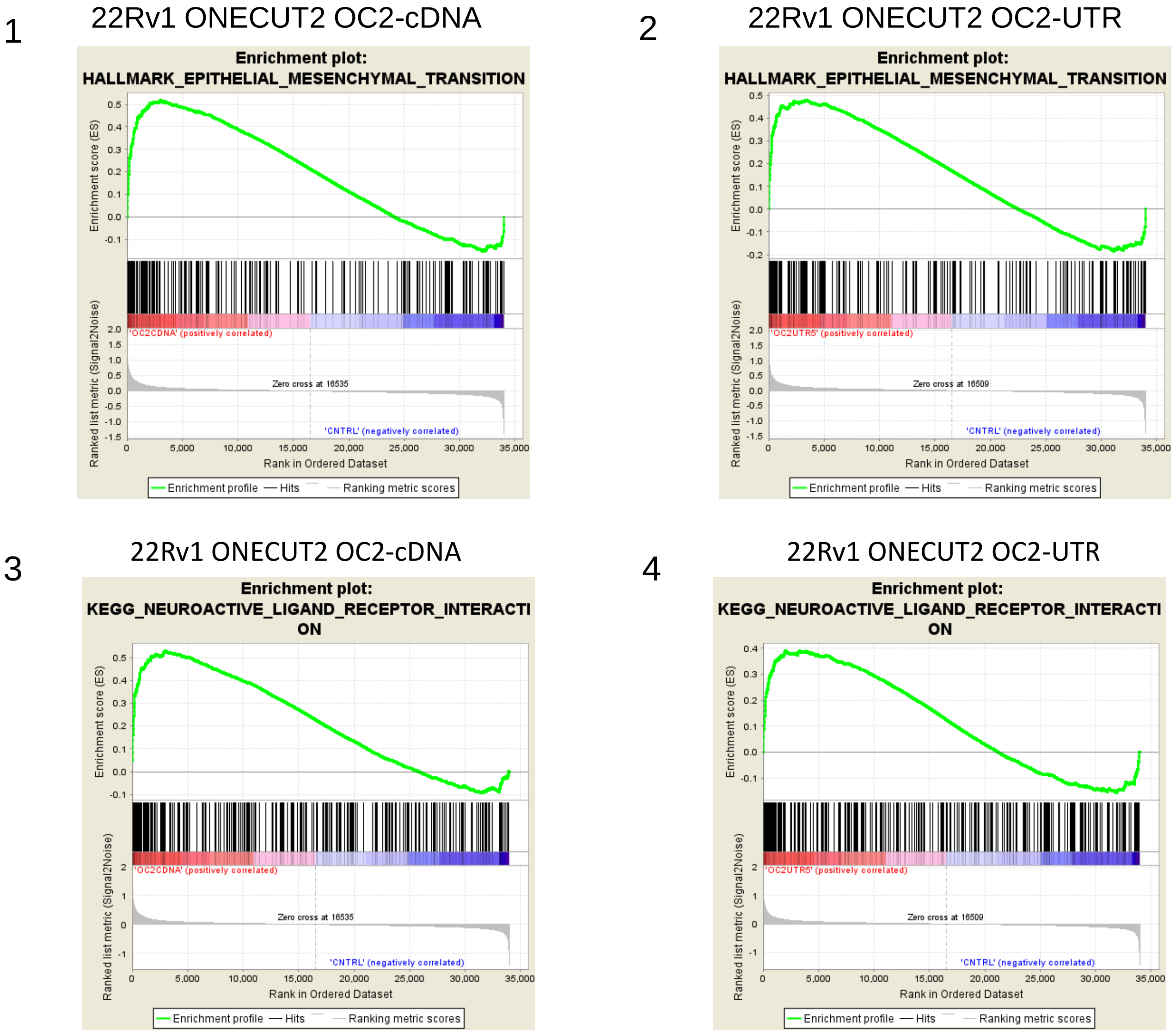

P

KEGG and Hallmarks GSEA Overlaps

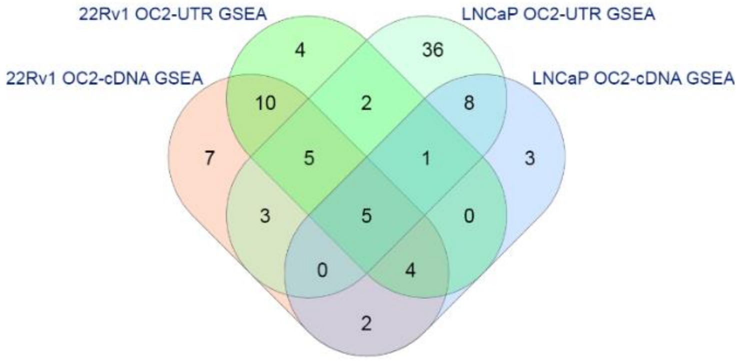

(O) (1 and 2) Gene Set Enrichment Analysis of OC2 protein and 3’ UTR both show enhanced activity EMT activity. (3 and 4) Gene Set Enrichment Analysis of OC2 protein and 3’ UTR show similar neuronal signaling activity. (P) A four-way Venn diagram of all LNCaP OC2cDNA/3’UTR and 22Rv1 OC2cDNA/3’UTR significantly enriched biological process and pathways.

Figure 4 Supplemental

Q

GSEAs in discordance between OC2 cDNA and OC2 3’ UTR

1

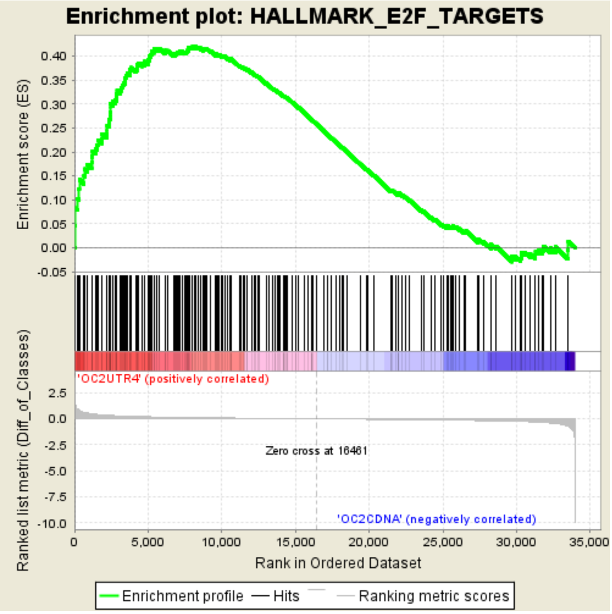

2

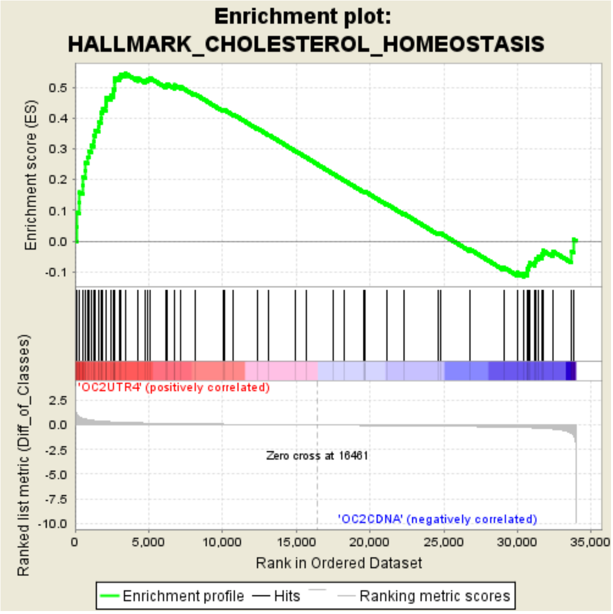

GSEAs in discordance between OC2 cDNA and OC2 3’ UTR

3

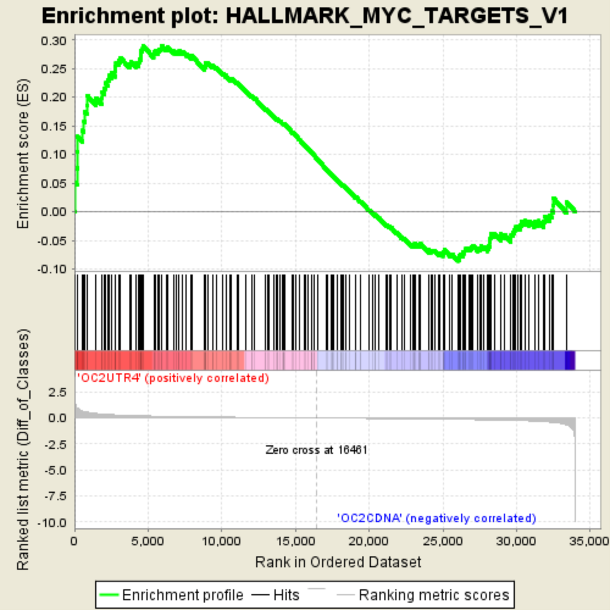

4

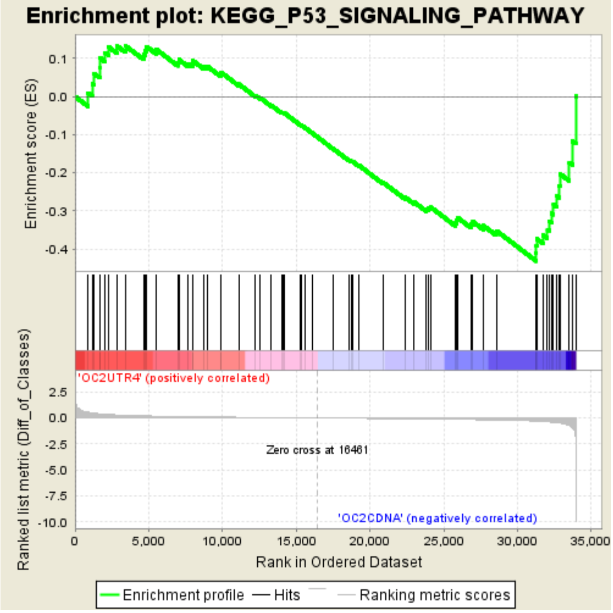

(Q) Important differences between ONECUT protein and 3‘ UTR network activated gene sets. (1) Gene Set Enrichment Analysis of OC2 protein and 3’ UTR show that OC2 3 ‘UTR has increased transcription factor E2F activity. (2) OC2 3’ UTR has significantly increased Cholesterol homeostasis activity. (3) OC2 3’ UTR shows significantly increased activity of MYC. (4) OC2 protein has increased p53 signaling activity that OC2 3’ UTR does not.

Figure 4 Supplemental

R

1 22Rv1 ONECUT2 OC2-cDNA

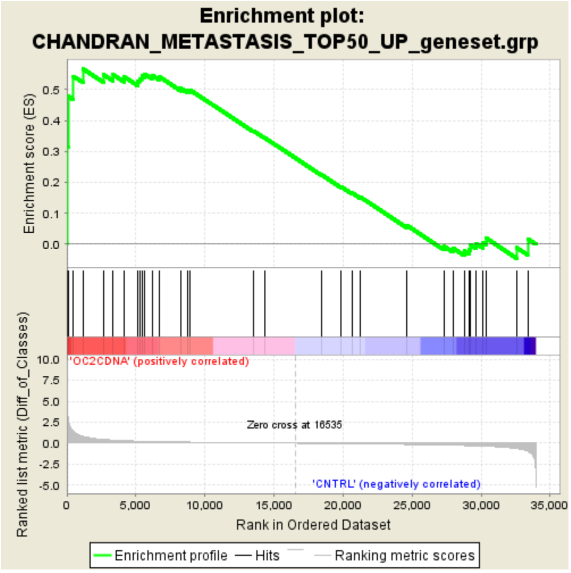

2 22Rv1 ONECUT2 OC2-UTR

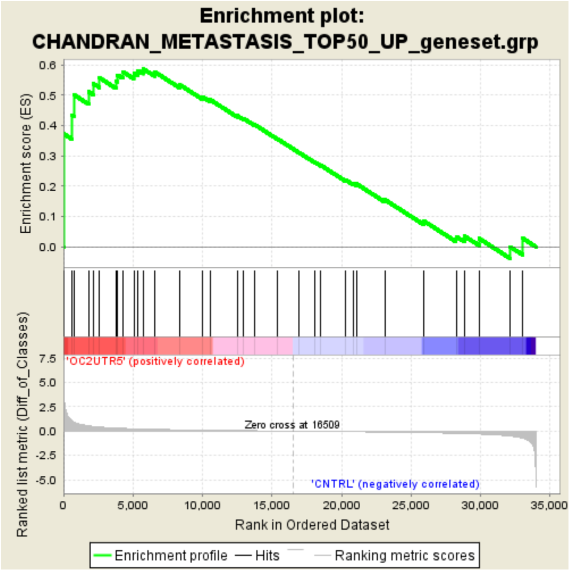

S

1 GSEAs of PRC/EZH2 OC2-cDNA

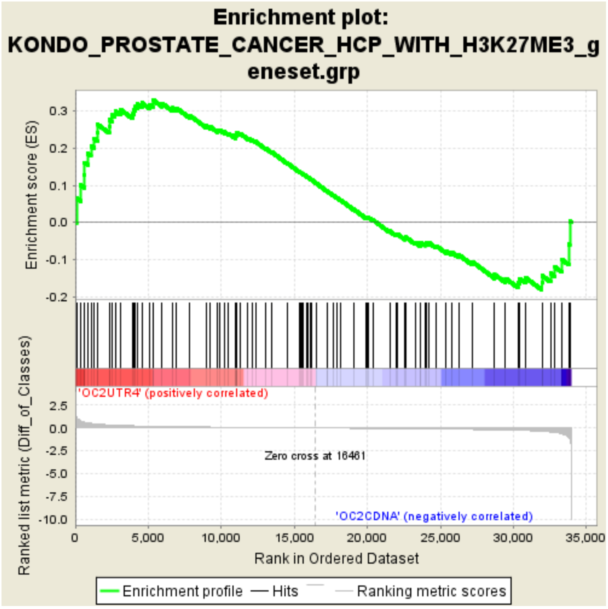

2 GSEAs of PRC/EZH2 OC2-UTR

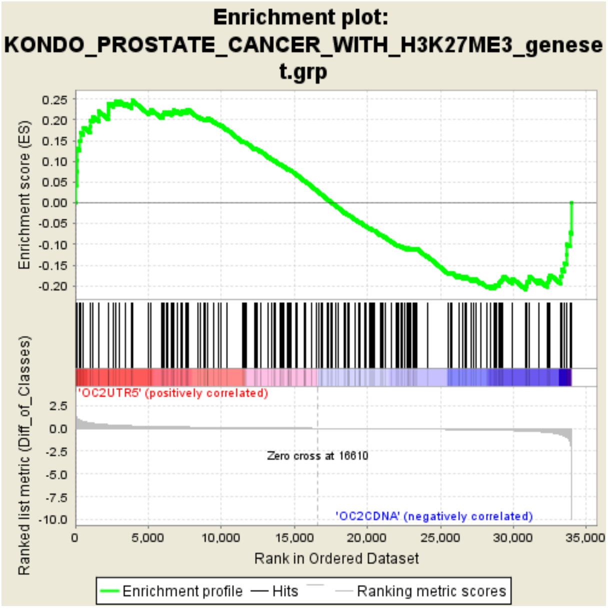

(R) (Panels 1 and 2) Gene Set Enrichment Analysis of OC2 protein and 3’ UTR show similar metastatic promoting network activity. (S) (Panels 1 and 2) Gene Set Enrichment Analysis of OC2 protein and 3’ UTR show network correlation with hypermethylation activity in prostate cancer.

Figure 4 Supplemental

T

1 GSEAs of PRC/EZH2 OC2-cDNA

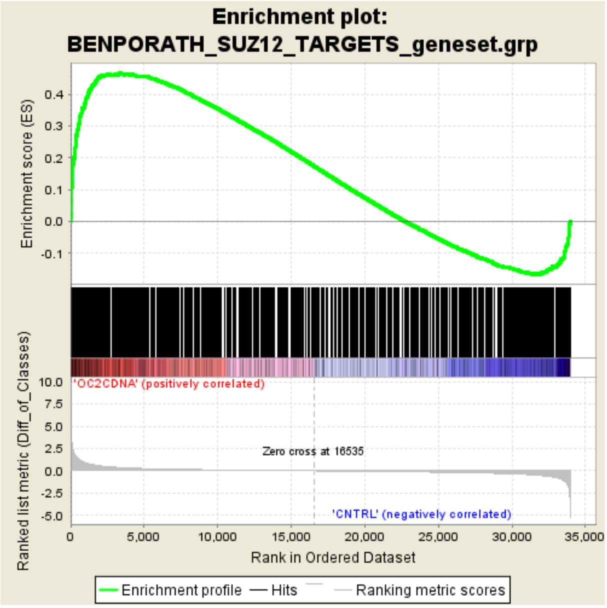

2 GSEAs of PRC/EZH2 OC2-UTR

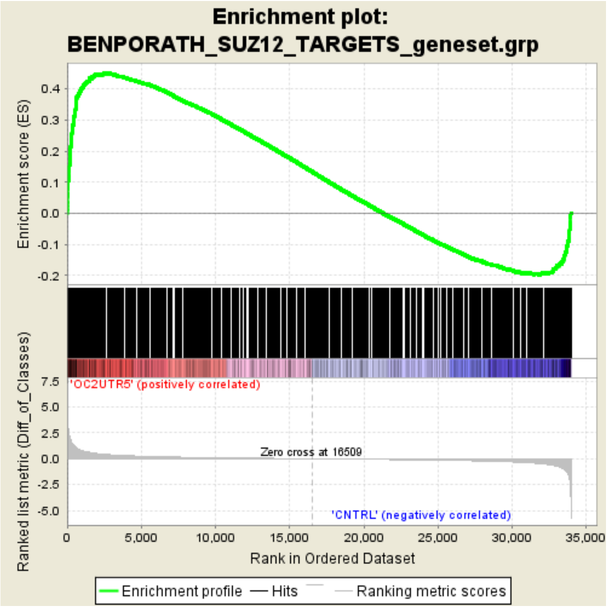

3 GSEAs of PRC/EZH2 OC2-cDNA

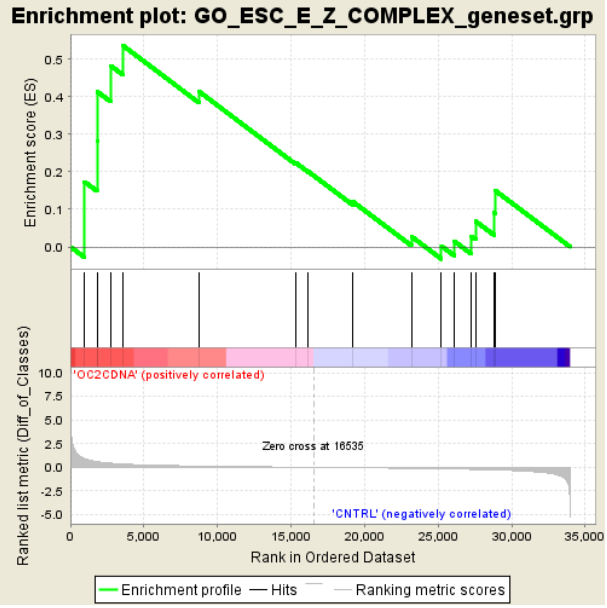

4 GSEAs of PRC/EZH2 OC2-UTR

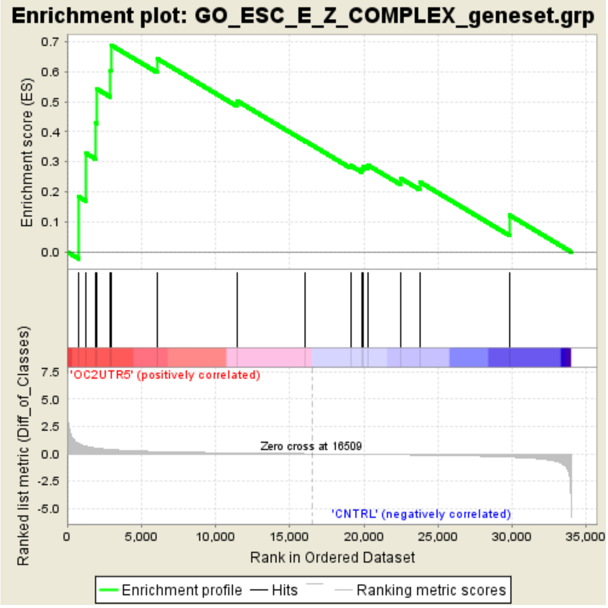

(T) Panels 1, 2, 3 and 4. Gene Set Enrichment Analysis of OC2 protein and 3’ UTR show similar significant enrichment of PRC activity.

Figure 5 Supplemental

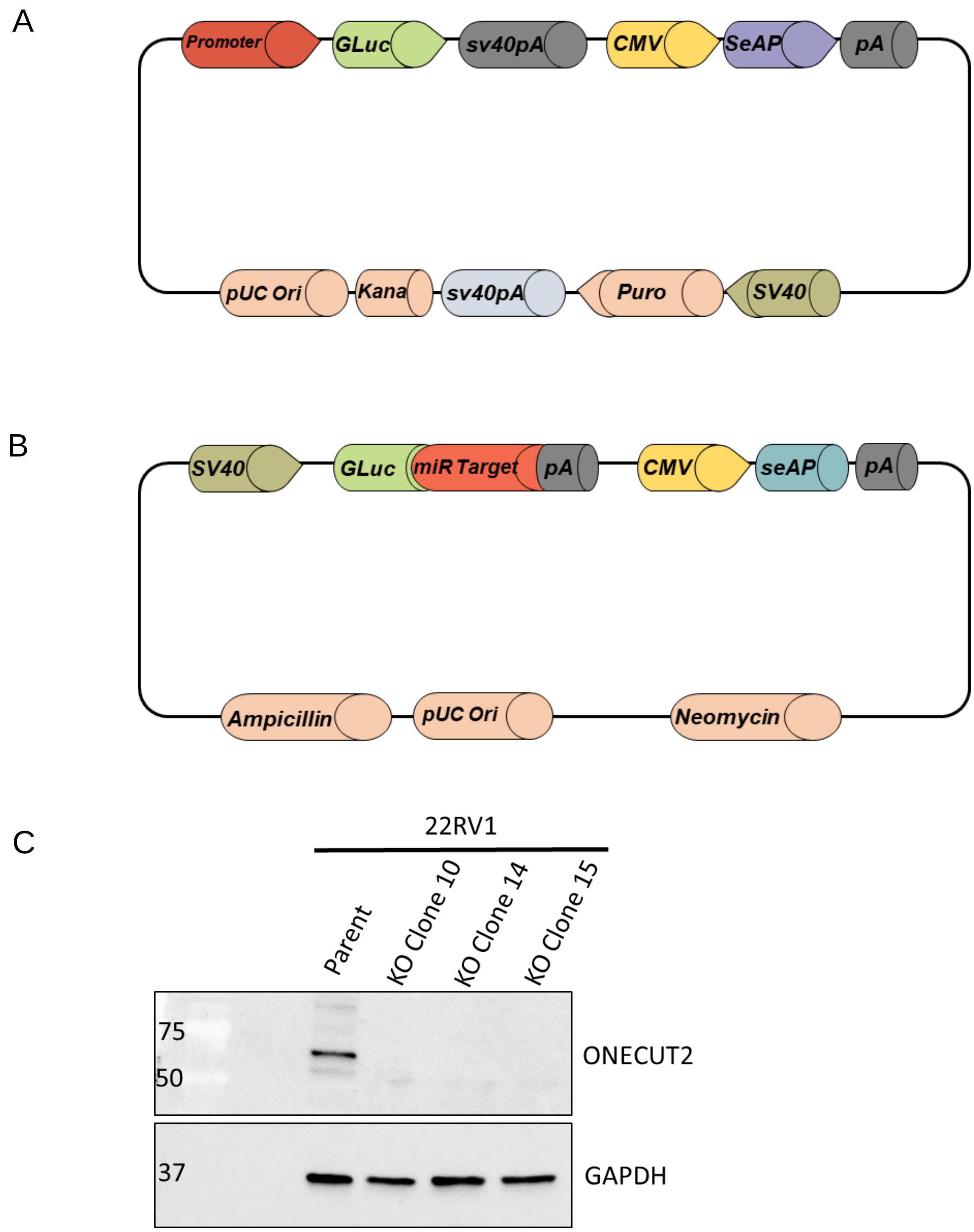

(A) Vector map of dual-luciferase promoter reporter. (B) Vector map of dual-luciferase 3' UTR reporter. (C) Capture of autoradiograph from Western blot showing ONECUT2 Protein in 22Rv1 CRISPR OC2 knockout clones.

Figure 8

A

Androgen Modulating lncRNAs	LNCAP OC2 UTR Log Fold Change	Adjusted P-Value	LNCAP OC2 cDNA Log Fold Change	Adjusted P-Value	22Rv1 OC2 UTR Log Fold Change	Adjusted P-Value	22Rv1 OC2 cDNA Log Fold Change	Adjusted P-Value	Reference
CTBP1-DT	-0.54	0.0005	-0.55	0.0002	0.41	0.006	0.36	0.033	PMID: 25552498
HOTAIR	0.86	0.0002	ns	ns	-0.64	0.003	-1.14	1.82E-05	PMID: 26411689
HOTAIRM1	-1.08	2.65E-07	ns	ns	-0.75	7.97E-06	-0.70	8.24E-05	PMID: 26411689
PCGEM1	-1.28	0.006	ns	nS	ns	ns	0.93	0.001	PMID: 27682980
DRAIC	-0.48	0.027	-0.68	0.001	-1.16	1.60E-09	-0.85	9.19E-07	PMID: 25700553
PlncRNA-1 (CBR3-AS1)	-1.28	3.39E-09	-0.44	0.001	-1.69	2.07E-10	-1.10	8.28E-07	PMID: 26808578
ARLNC1	-0.55	0.0001	-0.68	3.39E-06	ns	ns	ns	ns	PMID: 29808028
SOCS2-AS1	-0.82	0.040	ns	ns	ns	ns	ns	ns	PMID: 27342777

(A) Microarray gene expression data of lncRNAs known to support AR activity.
